# Inflammation modulates intercellular adhesion and mechanotransduction in human epidermis via ROCK2

**DOI:** 10.1016/j.isci.2023.106195

**Published:** 2023-02-14

**Authors:** Maria S. Shutova, Julia Borowczyk, Barbara Russo, Sihem Sellami, Justyna Drukala, Michal Wolnicki, Nicolo C. Brembilla, Gurkan Kaya, Andrei I. Ivanov, Wolf-Henning Boehncke

**Affiliations:** 1 University of Geneva, Department of Pathology and Immunology, Geneva, Switzerland; 2 University Hospitals of Geneva, Division of Dermatology and Venereology, Geneva, Switzerland; 3 Geneva Centre for Inflammation Research, Faculty of Medicine, University of Geneva, Geneva, Switzerland; 4 Jagiellonian University, Department of Cell Biology, Faculty of Biochemistry, Biophysics and Biotechnology, Cracow, Poland; 5 Department of Pediatric Urology, Jagiellonian University Medical College, Cracow, Poland; 6 Department of Inflammation and Immunity, Lerner Research Institute, Cleveland Clinic Foundation, Cleveland, OH, USA

**Keywords:** Biological sciences, Immunology, Immune response, Genomics

## Abstract

Aberrant mechanotransduction and compromised epithelial barrier function are associated with numerous human pathologies including inflammatory skin disorders. However, the cytoskeletal mechanisms regulating inflammatory responses in the epidermis are not well understood. Here we addressed this question by inducing a psoriatic phenotype in human keratinocytes and reconstructed human epidermis using a cytokine stimulation model. We show that the inflammation upregulates the Rho-myosin II pathway and destabilizes adherens junctions (AJs) promoting YAP nuclear entry. The integrity of cell-cell adhesion but not the myosin II contractility *per se* is the determinative factor for the YAP regulation in epidermal keratinocytes. The inflammation-induced disruption of AJs, increased paracellular permeability, and YAP nuclear translocation are regulated by ROCK2, independently from myosin II activation. Using a specific inhibitor KD025, we show that ROCK2 executes its effects via cytoskeletal and transcription-dependent mechanisms to shape the inflammatory response in the epidermis.

## Highlights

• Inflammation disrupts adherens junctions and enhances mechanosignaling in the epidermis
• These effects rely on the Rho-ROCK2 pathway but are myosin independent
• ROCK2 inhibitor KD025 partially rescues the keratinocyte inflammatory phenotype

## Graphical abstract

**Figure:**
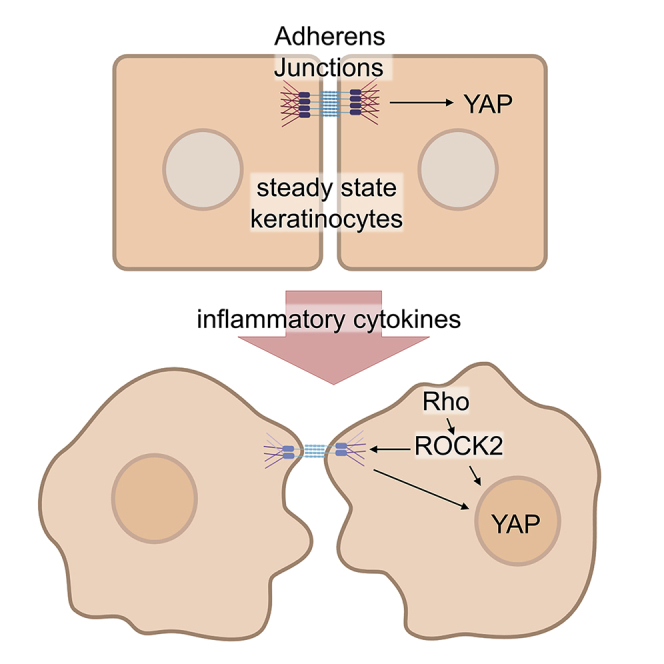

## Introduction

Inflammation in epithelial tissues has been associated with impaired permeability of epithelial layers. In simple columnar epithelia and vascular endothelium, the inflammatory stimuli increase paracellular permeability by inducing the reorganization of cell-cell contacts.^1^^,^^2^^,^^3^^,^^4^^,^^5^^,^^6^ In contrast, the compromised epidermal barrier in inflamed skin, such as in psoriasis and atopic dermatitis, is explained mainly by the improper keratinocyte differentiation, sphingolipid synthesis, and, as a result, the malformation of the cornified layer in the multi-layered epidermis.^7^^,^^8^^,^^9^^,^^10^^,^^11^^,^^12^^,^^13^

The keratinocyte cell-cell adhesions have a crucial role for the functional integrity and mechanical properties of the epidermis. Among them, E-cadherin-based adherens junctions (AJs) are the most universal and dynamic, and their dysfunction results in disassembly of other junctional complexes. Although some data point toward the role of cell-cell junctions (mainly tight junctions) in the epidermal barrier regulation,^14^^,^^15^^,^^16^^,^^17^^,^^18^ the role of epidermal AJs in the pathogenesis of cutaneous inflammation has not been studied systematically. A defective AJ assembly might be, indeed, an emerging mechanism for increased permeability in inflamed skin. For instance, a widening of the intercellular spaces and a decreased expression of AJ proteins have been described in psoriatic and atopic epidermis suggesting alterations in cell-cell adhesion.^19^^,^^20^^,^^21^^,^^22^^,^^23^^,^^24^

The second aspect that links AJs to the inflammation pathogenesis is the fact that AJs represent “hot spots” for mechanotransduction.^25^^,^^26^^,^^27^ An upregulation of mechanotransduction pathways is considered a crucial factor in the development of human disorders such as cancer, fibrosis, cardiovascular, and inflammatory conditions, where the abnormal mechanosignaling can lead to increased cell proliferation, motility, deposition of extracellular matrix, and improper cell differentiation.^28^^,^^29^^,^^30^ Mechanoresponsive transcriptional co-activator yes-associated protein (YAP) and its paralog TAZ (transcriptional co-activator with PDZ-binding motif, also known as WW Domain Containing Transcription Regulator 1, WWTR1) play a central role in the regulation of proliferation and differentiation in multiple cell types,^31^^,^^32^^,^^33^ including epidermal stem cells.^34^^,^^35^^,^^36^ The Hippo pathway controls YAP signaling by restricting YAP nuclear translocation and is, in turn, coordinated via multiple mechano- and chemo-dependent upstream mechanisms, such as cell adhesion, shape and polarity, matrix stiffness, and actin-myosin contractility, as well as soluble extracellular mediators, growth factors, and cytokines.^33^^,^^37^^,^^38^ In particular, the YAP pathway can mediate the contact inhibition of cell proliferation in epithelia, and it appears to be negatively regulated by stable cell-cell adhesions, also restricting the activation of the AJ scaffolding protein β-catenin and the Wnt signaling.^39^^,^^40^^,^^41^^,^^42^ Several studies have shown an increase in expression and/or nuclear localization of β-catenin in epithelial cells indicating activation of canonical Wnt signaling**,** which correlated with nuclear translocation of YAP/TAZ in mouse models of colitis and corneal inflammation.^42^^,^^43^^,^^44^ In the context of skin inflammation, however, there is no consensus on the role of β-catenin and its indispensability for the epidermal homeostasis.^22^^,^^45^^,^^46^^,^^47^^,^^48^^,^^49^^,^^50^

The cell adhesion structures and adhesion-mediated mechanosignalling are globally regulated by the actin-myosin cytoskeleton. Nonmuscle myosin II (NMII) is enriched at the perijunctional actin belt, and a proper level of its activity is necessary for the AJ assembly and integrity. Actin-myosin contractility can be beneficial for the paracellular barrier tightness in endothelium and intestinal epithelium.^51^^,^^52^^,^^53^^,^^54^ On the other hand, a large body of data indicate that the barrier breakdown is driven by increased NMII contractility and the reorganization of perijunctional actin during intestinal and bronchial inflammation.^6^^,^^55^^,^^56^^,^^57^^,^^58^^,^^59^^,^^60^^,^^61^ Activation of the myosin light chain (MLC) kinases (either MLCK or Rho-associated kinase, ROCK) has been linked to colon inflammation in patients and murine models of colitis,^6^ as well as to the endothelial hyperpermeability.^60^^,^^62^^,^^63^^,^^64^^,^^65^^,^^66^ However, specific roles of MLC kinases and actin-myosin contractility in inflammation-induced AJ disassembly remain incompletely understood^4^ and have not been demonstrated in epidermal inflammation.

In addition to the AJ regulation, the activation of Rho-ROCK-NMII pathway can also promote YAP nuclear translocation and the downstream gene transcription.^31^^,^^67^^,^^68^ In recent years, the distinct roles of two kinases ROCK1 and ROCK2 downstream of Rho in the epithelial cell adhesion, proliferation, and differentiation began to emerge.^69^^,^^70^^,^^71^ Moreover, studies have reported a positive feedback regulation of YAP by ROCK2^72^ and association of ROCK2 overexpression with cell hyperproliferation and cancer progression, presumably via regulating cell contractility.^73^

To the present date, the regulation and interconnection of cell-cell adhesion, cell contractility, and the downstream mechanosignaling in inflamed epidermis remain unclear.^13^ At the same time, innovative topical therapies for chronic inflammatory skin conditions such as psoriasis are much needed.^74^ In this study, we have modeled psoriatic inflammation using a specific cytokine stimulation^75^ of non-differentiated human keratinocytes and reconstructed human epidermis (RHE), which closely reproduces epidermal differentiation and stratification processes. We show that inflammatory conditions disrupt AJs, increase epidermal permeability, and induce activation of Rho signaling and nuclear translocation of YAP. By using a specific ROCK2 inhibitor KD025 (SLx-2119),^76^ we reveal a ROCK2-dependent but contractility-independent way of regulation of inflammatory mechanoresponse in human epidermis.

## Results

### Adherens junctions are disrupted in M5-stimulated keratinocytes

To analyze the regulation of epidermal AJs during psoriatic inflammation in a model *in virto* system, primary juvenile human keratinocytes and N/TERT immortalized normal human keratinocytes^77^ were grown either as monolayers (in 2D conditions, without differentiation) or as multi-layered fully differentiated RHE (see Methods). The use of keratinocyte monolayers gives technical advantages for imaging analysis of the cell adhesions and cytoskeletal organization and puts our experiments in the broad context of previous studies that investigated keratinocyte junctions in cell monolayers. On the other hand, the more complex RHE cultures with fully differentiated epidermal cell layers recapitulate better the *in vivo* skin biology and pathophysiology.

To induce a psoriatic phenotype *in vitro*, we stimulated the cultures with a cocktail of specific inflammatory cytokines (here called M5), as previously described.^75^ The mix included interleukin (IL)-17A, IL-1α, oncostatin M, TNFα, and IL-22 at concentration 10 ng/mL each. The treatment of keratinocytes with this cytokine cocktail induced a strong psoriasis-like inflammatory response characterized by the production of inflammatory mediators IL-36γ, IL-8, and Chemokine (C-C motif) ligand 20 (CCL20) (Figure S1A) and a loss of the keratinocyte differentiation marker cytokeratin-10 in stratified RHEs (Figure S1B).

**Figure 1:**
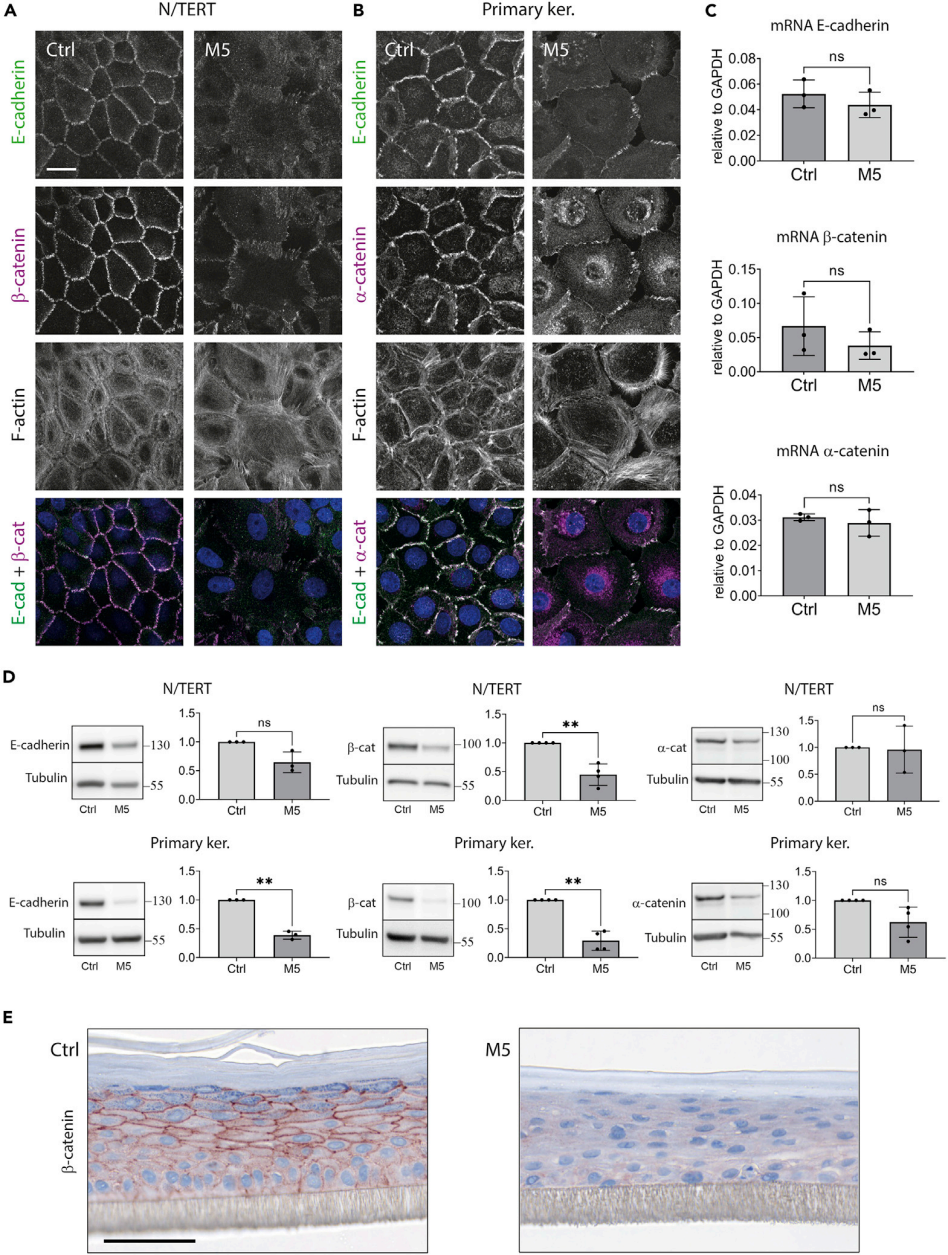
AJs in keratinocytes are affected by the inflammatory stimuli (A and B) Immunofluorescence staining for AJ proteins and F-actin in non-differentiated N/TERT (A) or primary keratinocytes (B) after 48 h of M5 stimulation. Maximum projections of four apical confocal slices are shown. Scale bar, 20 μm. (C) mRNA for AJ proteins in RHEs from N/TERT keratinocytes after 24 h of M5 stimulation, data from three independent experiments. Paired t-test was applied. GAPDH is a stable housekeeping gene in keratinocytes, whose expression level does not change in the experiments with cytokine treatments.^78^ (D) Western blotting for AJ proteins in RHEs after 48 h of M5 stimulation. Data from independent experiments were normalized to the respective α-tubulin loading control, and the band intensities were presented as arbitrary units. Mean ± SD. Paired t-test, ∗∗p < 0.01. (E) Immunohistochemical staining for β-catenin in RHEs from primary keratinocytes after 48 h of M5 stimulation. Scale bar, 50 μm.

The most prominent M5-induced phenotype in monolayer cell cultures was an increased cell spreading area and a formation of gaps between the cells within keratinocyte islands. Similarly, increased intercellular gaps were frequently seen in the cytokine-stimulated RHEs. Indeed, we observed a dramatic reorganization of AJ structures and the associated actin cytoskeleton, which predominantly formed radial fibers instead of tangential actin bundles in the perijunctional area (Figures 1A and 1B). Moreover, we observed a decreased localization of E-cadherin, α-catenin, and β-catenin to AJs and a lower protein, but not mRNA, expression of E-cadherin and β-catenin, which was most prominent in RHEs after 48 h of the cytokine stimulation (Figures 1C–1E). We did not detect nuclear localization of β-catenin in any of the samples.

### M5 cytokine stimulation increases intracellular contractility in monolayer cultures but not in RHEs

**Figure 2:**
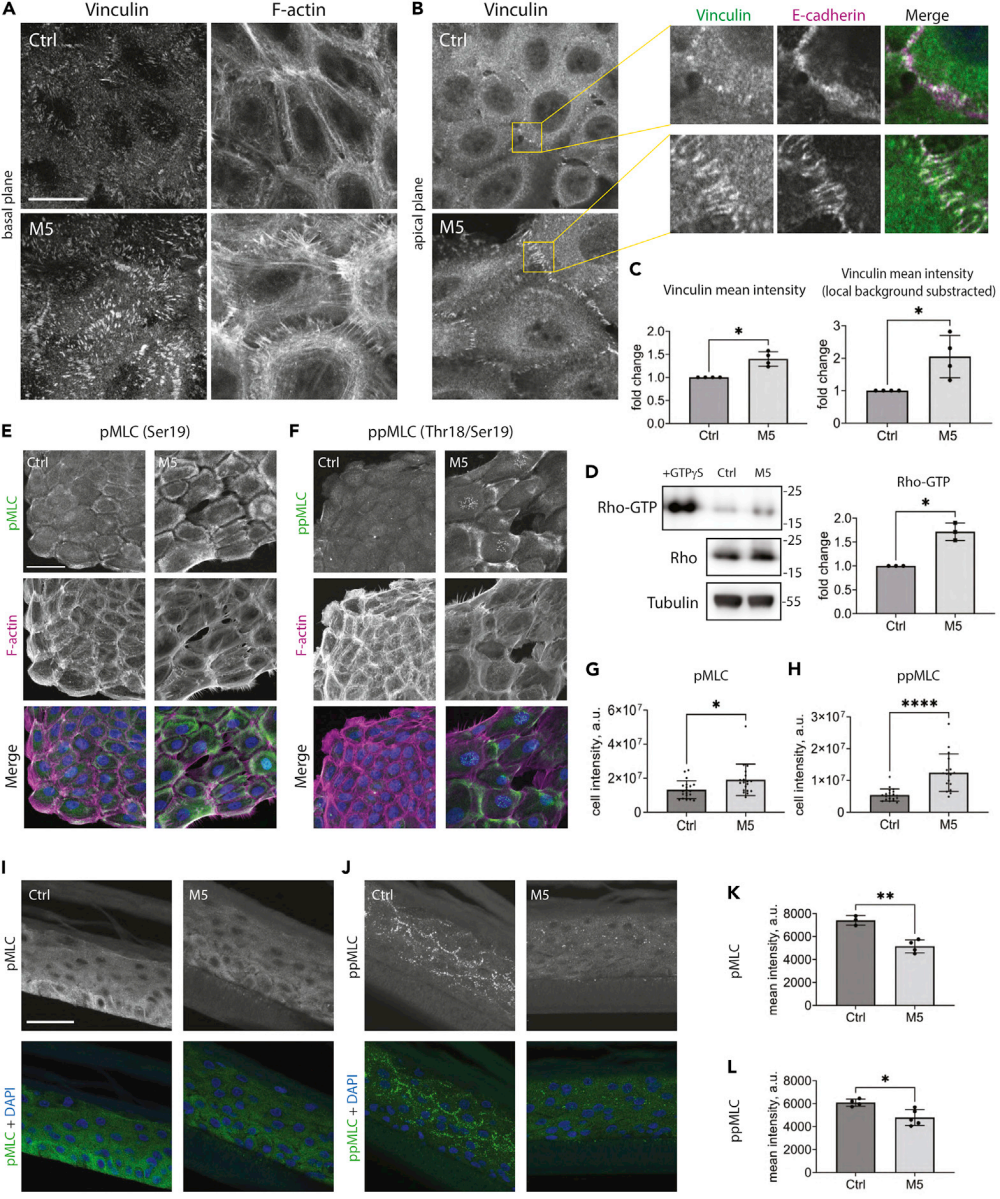
M5 cytokine stimulation differentially affects MLC activation in monolayer and RHE cultures (A) Immunofluorescence staining for vinculin at focal adhesions in M5-stimulated N/TERT, single confocal slice in the basal plane. (B) Immunofluorescence staining for vinculin at the AJs in M5-stimulated N/TERT, single confocal slice in the apical plane. (C) Quantitative analysis of the vinculin recruitment to AJs shown in (B), data from four independent experiments. Mean ± SD. Paired t-test, ∗p < 0.05. (D) Western blotting for active Rho in M5-stimulated non-differentiated N/TERT. One representative experiment out of three independent experiments is shown. Mean ± SD. Paired t-test, ∗p < 0.05. (E and F) Immunluorescence staining for pMLC (E) and ppMLC (F) in non-differentiated N/TERT, confocal max projections. One representative experiment of three is shown. (G and H) Quantification of pMLC (G) and ppMLC (H) fluorescence intensity per cell for non-differentiated N/TERT. Mean ± SD. Unpaired t-test, ∗p < 0.05, ∗∗∗∗p < 0.0001. (I and J) Immunofluorescence staining for pMLC (I) and ppMLC (J) in RHEs from N/TERT, confocal max projections. (K and L) Quantification of pMLC (K) and ppMLC (L) mean fluorescence intensity per field of view in RHEs from N/TERT cells. Mean ± SD. Unpaired t-test, ∗p < 0.05, ∗∗p < 0.01. The stimulation with M5 was done for 24 h in all panels. Scale bars, 20 μm (A and B), 50 μm (E, F, I, and J).

We hypothesized that the changes in the actin cytoskeleton and the formation of the intercellular gaps may indicate an increased cellular contractility. Indeed, we observed an increase in the formation of ventral stress fibers and the size of focal adhesions in M5-stimulated cells in 2D cultures (Figure 2A). The analysis of tension-sensitive vinculin recruitment to AJs^27^ revealed a higher vinculin recruitment to the AJs following M5 stimulation (Figures 2B and 2C). Accordingly, the stimulation increased the activation of Rho-GTPase (Figure 2D) and NMII, reflected by the mono- (Ser19) and double (Thr18/Ser19)-myosin light chain (MLC) phosphorylation in both N/TERT (Figures 2E–2H) and primary (Figure S2) keratinocytes.

However, in RHE cultures, the phospho-Ser19 (pMLC) and phospho-Thr18/Ser19 (ppMLC) immunolabeling patterns appeared to be different from those observed in monolayer keratinocytes. In control RHE cultures, the pMLC signal was distributed throughout the cell cytoplasm and had a higher intensity in the basal cell layer, whereas ppMLC mostly labeled cell borders in the spinous and granular layers. Surprisingly, the stimulation of RHEs with M5 cytokines decreased labeling intensity for both pMLC and ppMLC, which is in contrast with the effect in monolayer cultures (Figures 2I–2L).

### M5 cytokine stimulation induces YAP nuclear translocation

**Figure 3:**
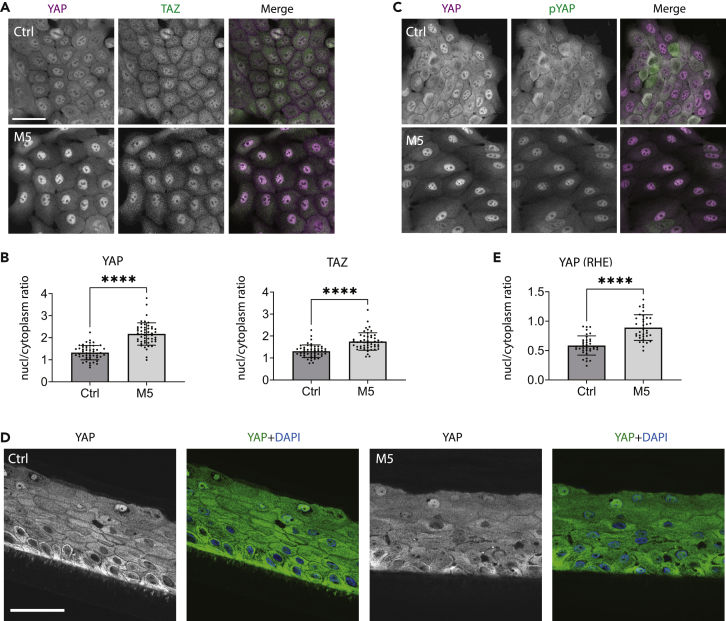
YAP nuclear targeting is increased in response to M5 cytokine stimulation (A and B) Immunofluorescence staining for YAP/TAZ, confocal maximum projection (A), and quantification of their nucleus-to-cytoplasm ratio (B) in non-differentiated N/TERT after 24 h of M5 stimulation. One representative experiment of four is shown. See also Figure S3. Mean ± SD. Unpaired t-test, ∗∗∗∗p < 0.0001. (C) Immunofluorescence staining for YAP and phosphorylated YAP (pYAP) in non-differentiated N/TERT after 24 h of M5 stimulation, confocal maximum projections. One representative experiment of two is shown. (D) Immunofluorescence staining of YAP, single confocal slice. (E) Quantification of YAP nucleus-to-cytoplasm ratio (see STAR Methods) in RHEs from N/TERT after 24 h of M5 stimulation. One representative experiment is shown. Mean ± SD. Unpaired t-test, ∗∗∗∗p < 0.0001. Scale bars, 50 μm.

Given the effects of M5 cytokines on the NMII activation, we investigated if the mechanotransduction in keratinocytes was affected by these inflammatory stimuli. Specifically, the effect of the M5 stimulation on the nuclear translocation of a major mechanosensitive co-activator of transcription YAP and its homolog TAZ was analyzed. In cultured keratinocytes, YAP was present in both nuclei and cytoplasm, showing a trend toward more nuclear distribution in the peripheral cells of the island and more cytoplasm-enriched in the densely packed cells in the center of the island. Cytokine stimulation for 24 h increased the nucleus-to-cytoplasm ratio of YAP and TAZ in most cells within islands (Figures 3A, 3B, and S3). Moreover, it also decreased the inactivating phosphorylation of YAP at Ser127 (Yu et al., 2015) (Figure 3C).

In control RHEs, the pattern of YAP subcellular localization resembled one of the normal human epidermis where YAP was enriched in the nuclei of basal keratinocytes and excluded from the nuclei in the differentiated cells of other layers, except individual cells in the top cornified layer.^79^ Overall, the YAP nucleus-to-cytoplasm ratio was substantially lower in RHEs than in monolayer cultures. Similarly to the cells in monolayers, YAP nuclear translocation in the RHEs was induced following cytokine stimulation (Figures 3D and 3E).

We hypothesized that the differences in MLC activation and YAP nuclear targeting in monolayers versus RHE cultures may at least partially be explained by the differences in the stiffness of the cell environment. Whereas monolayer cultures are supported by the rigid substrate (approximately 60 GPa for glass), the cells within the stratified epidermal layer are engaged by a much softer environment of lower cell layers (at 5–15 kPa range). To test the role of the substrate rigidity in the inflammation-induced phenotypes, we plated N/TERT keratinocytes onto the hydrogels of various stiffness, coated with collagen IV (Figure S4). Control cells formed smaller colonies while grown on very soft hydrogels (0.5 kPa) compared with colonies on the 12 or 50 kPa substrates. We also observed a trend for more cytoplasmic YAP localization but higher overall expression with the decrease of substrate stiffness. This could explain the observed differences between the average YAP nucleus-to-cytoplasm ratio numbers in control monolayer cultures and control RHEs.

M5 stimulation strongly induced YAP nuclear translocation on all substrates (Figures S4A and S4B). Surprisingly, the activation of NMII contractility via MLC phosphorylation appeared to be higher in the colonies grown on softer matrices but was similarly upregulated by the cytokine stimulation (Figure S4C). Moreover, the control cells grown on the physiological range of stiffness exhibited different pattern of ppMLC at the edge and in the center of large colonies (Figure S4D). The central cells that were densely surrounded by neighboring cells displayed prominent accumulation of ppMLC at cell-cell junctions. We conclude that the substrate stiffness sensed through focal adhesions can be only one of the factors defining the intracellular contractile forces developed by the keratinocytes and the YAP nuclear targeting, whereas, the cell-cell interactions between keratinocytes and/or their differentiation status within colonies or stratified layers may have prevailing effect on YAP signaling.

### Inhibition of ROCK2 but not ROCK1 and ROCK2 together reverts cytokine-induced effects on AJs and YAP

Because we observed similar effects of the M5 stimulation on primary keratinocytes and N/TERT cell line, we decided to use the N/TERT cells in the subsequent functional experiments.

**Figure 4:**
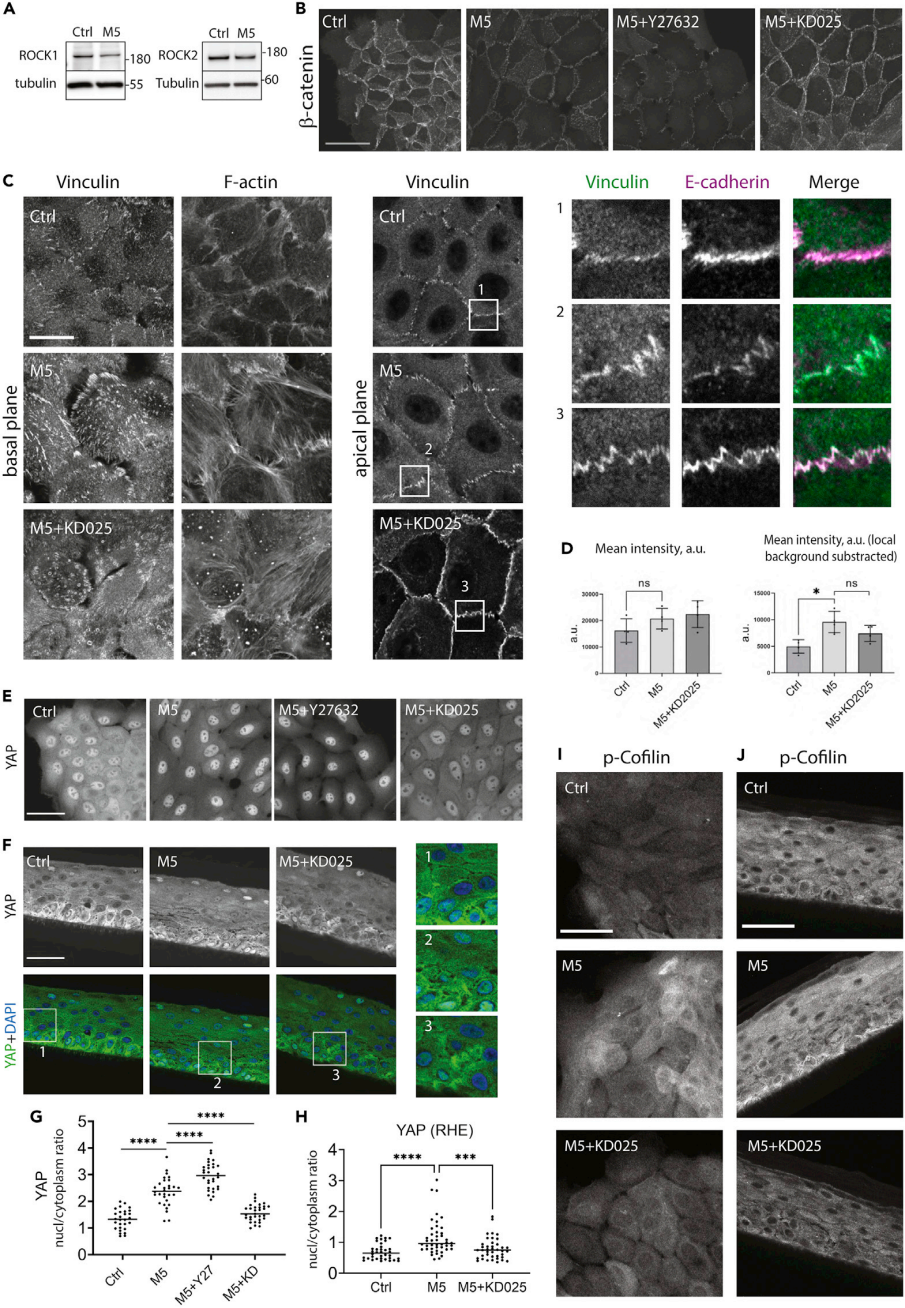
The effect of ROCK inhibition on M5-stimulated N/TERT keratinocytes (A) Western blotting of ROCK1 and ROCK2 expression in non-differentiated cells with and without M5 stimulation. (B) Immunofluorescence staining of β-catenin in indicated conditions, confocal max projections. (C) Immunofluorescence staining for vinculin at the focal adhesions (basal confocal slice) and the AJs (apical confocal slice) in cells stimulated for 24 h. Zoomed images show vinculin co-localization with E-cadherin. (D) Quantitative analysis of the vinculin recruitment to AJs shown in (C), data from four independent experiments. Mean ± SD. Paired t-test was applied. (E) Immunofluorescence staining of YAP in indicated conditions, confocal max projections. (F) Immunofluorescence staining of YAP in RHEs in indicated conditions, the brightest confocal slice. (G) Quantification of nucleus-to-cytoplasm ratio of YAP from (E), one representative experiment of three is shown. (H) Quantification of the nucleus-to-cytoplasm ratio of YAP in RHEs from (F). Mean ± SD. Unpaired t-test, ∗∗∗p < 0.001, ∗∗∗∗p < 0.0001. (I and J) Immunofluorescence staining of phosphorylated cofilin in indicated conditions in monolayer (I) and RHE (J) cultures. Scale bars, 50 μm (B, E, F, and J), 20 μm (C).

To understand the role of Rho-NMII pathway activation in the keratinocyte inflammatory response, we applied a small-molecule inhibition approach for the acute inhibition of MLC kinases ROCK1 and ROCK2, which were both expressed in our experimental system (Figure 4A). Pan-ROCK inhibitor Y-27632 applied simultaneously with the cytokine stimulation caused a complete loss of ppMLC from the stress fibers in M5-stimulated keratinocytes in monolayer cultures (Figure S5A) and further increased the M5-induced AJ disassembly (Figure 4B). In contrast, ROCK2-specific inhibitor KD025 caused only a partial loss of ppMLC in M5-stimulated cells in monolayer cultures (Figure S5A) and did not significantly affected the ppMLC level in stimulated RHEs (Figures S5B and S5C). Surprisingly, the inhibition of ROCK2 prevented the M5-induced disruption of the cell-cell contacts and the rearrangement of the perijunctional actin cytoskeleton from tangential to radial bundles (Figures 4B, S4D, and S4E). Moreover, the tension-sensitive vinculin recruitment to the sites of AJs was not decreased after ROCK2 inhibition in the M5-stimulated cells (Figures 4C and 4D). Remarkably, we found that KD025 treatment was able to retain YAP in the cytoplasm of the M5-stimulated keratinocytes, both in monolayers and in RHEs (Figures 4E–4H).

The aforementioned data suggest that ROCK2 inhibition partially rescued the M5-induced AJ and YAP phenotypes without affecting MLC phosphorylation. We hypothesized that the inhibition of ROCK2 by KD025 may exert its effects via NMII-independent mechnaisms. To test this idea, we treated keratinocytes with blebbistatin, which inhibits NMII motor activity. Blebbistatin treatment of control keratinocytes for 1 h caused disassembly of AJs and increased YAP nuclear translocation compared with the vehicle-treated cells (Figures S5F and S5G). By contrast, treatment of control keratinocytes with KD025 alone did not affect AJ integrity and cellular localization of YAP. Remarkably, when blebbistatin and KD025 were applied together, the blebbistatin-induced AJs disassembly and nuclear translocation of YAP were strongly inhibited (Figures S5F and S5G). These data strongly suggest that ROCK2 can regulate the AJ integrity and/or YAP mechanosignaling in an NMII-independent manner. Specifically, the cytokine-induced AJ disassembly can be mediated through the ROCK2-dependent altered actin filament dynamics. Indeed, a ROCK target LIM-kinase (LIMK) is known to inhibit the members of actin-depolymerizing factor/cofilin family via phosphorylation, and we found an increase of phosphorylated cofilin level associated with the cytokine stimulation and its subsequent decrease after ROCK2 inhibition (Figures 4I and 4J).

### ROCK2 inhibition partially rescues the cytokine-induced disruption of barrier in keratinocyte layers

**Figure 5:**
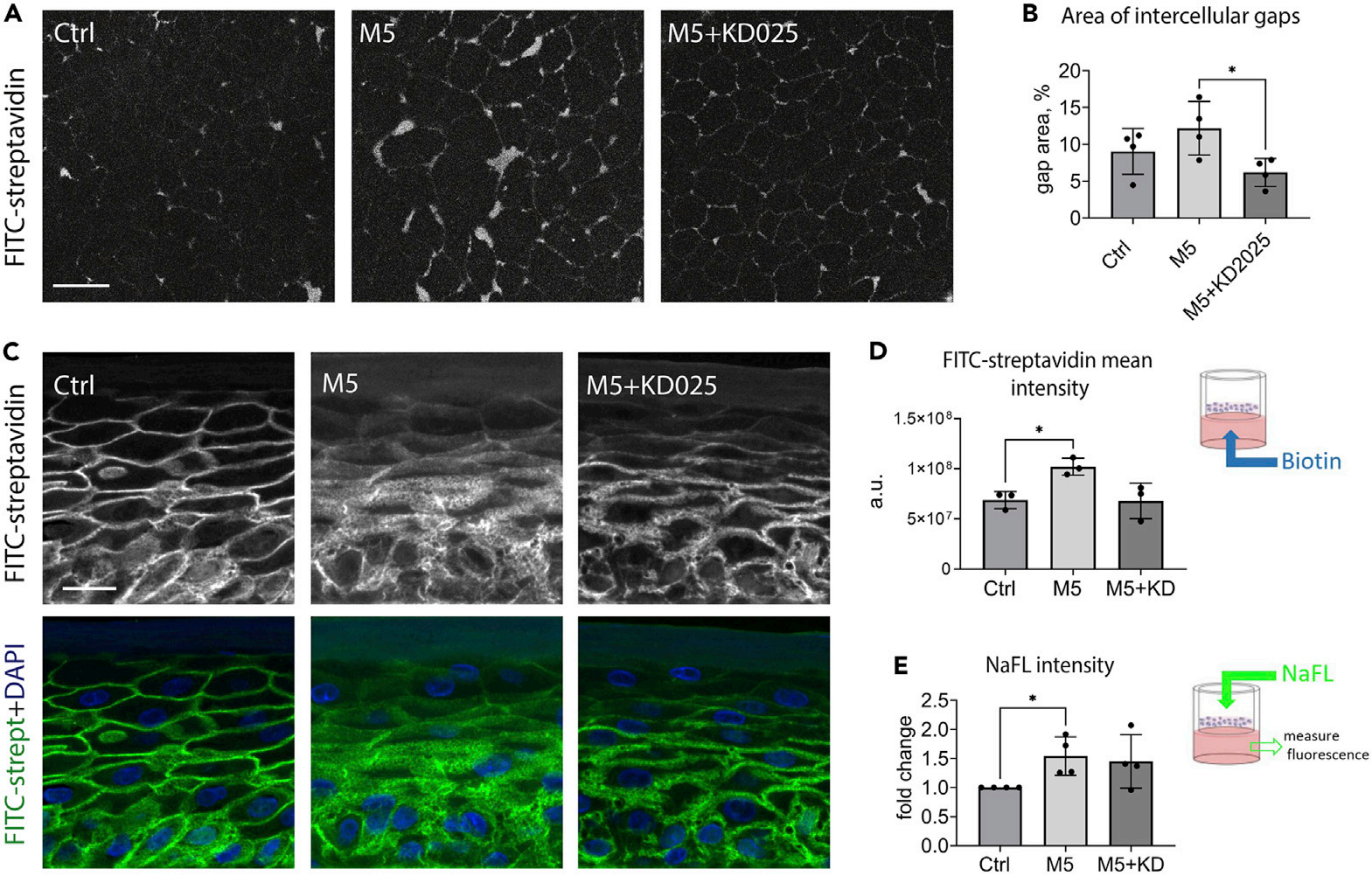
The effects of M5 and KD025 stimulation on the permeability of keratinocyte layers (A and B) Permeability assay in N/TERT monolayers: confocal images (maximum projections) of FITC-streptavidin revealing free biotinylated substrate (A) and quantification of the surface area of intercellular gaps (B). Mean ± SD. Paired t-test, ∗p < 0.05. (C and D) Basal-to-apical permeability assay: FITC-streptavidin revealing penetration of biotin in RHEs in indicated conditions (C, confocal sum slices) and the quantification of its intensity (D). Data from three independent experiments. Mean ± SD. Paired t-test, ∗p < 0.05. (E) Apical-to-basal Na-Fluorescein permeability assay on RHE cultures, data from four independent experiments. Mean ± SD. Paired t-test was applied. Scale bars, 50 μm (A), 20 μm (C).

The impaired barrier function of the inflamed epidermis is one of the clinically relevant consequences of skin inflammation. We tested whether the paracellular barrier is disrupted by proinflammatory cytokines in our experimental systems and whether pharmacological inhibition of ROCK2 that rescues AJs disassembly could also prevent the cytokine-induced barrier disruption. Our 2D permeability assay was adapted from a vascular permeability assay kit, which is based on detecting the gaps between cells grown in monolayers on the biotinylated substrate using fluorescein isothiocyanate (FITC)-streptavidin. As expected, the intercellular gap area increased after 24 h of M5 stimulation. The keratinocytes treated with both M5 and KD025 displayed smaller intercellular gaps indicating attenuation of barrier disruption (Figures 5A and 5B).

The permeability of RHEs was analyzed by adding biotin to the culture medium to allow its bottom-to-top penetration for 1 h before the sample fixation. The intercellular gaps were revealed by staining the sections with fluorescein-streptavidin. We observed widened intercellular spaces in RHEs after 48 h of the cytokine stimulation and a partial reversion of this phenotype in the presence of the ROCK2 inhibitor, especially in the granular layer (Figures 5C and 5D). Next, we tested the apical-to-basal permeability of the epidermal layers. Sodium fluorescein solution was applied on top of the dry cornified layer in RHEs after 24 h of stimulation, and the fluorescence in the bottom chamber was measured 24 h later. We observed an increased sodium fluorescein penetration upon the cytokine stimulation; however, KD025 treatment did not significantly affect the tracer permeability (Figure 5E).

Together, these data suggest that ROCK2 inhibition by KD025 improved AJ integrity and AJ-dependent paracellular barrier under inflammatory conditions but had no significant effect on the outermost epidermal barrier, which is largely dependent on the integrity of the cornified layer formed by terminally differentiated and dead keratinocytes.

### ROCK2 inhibition affects the cytokine-driven inflammatory molecular signature in keratinocytes

**Figure 6:**
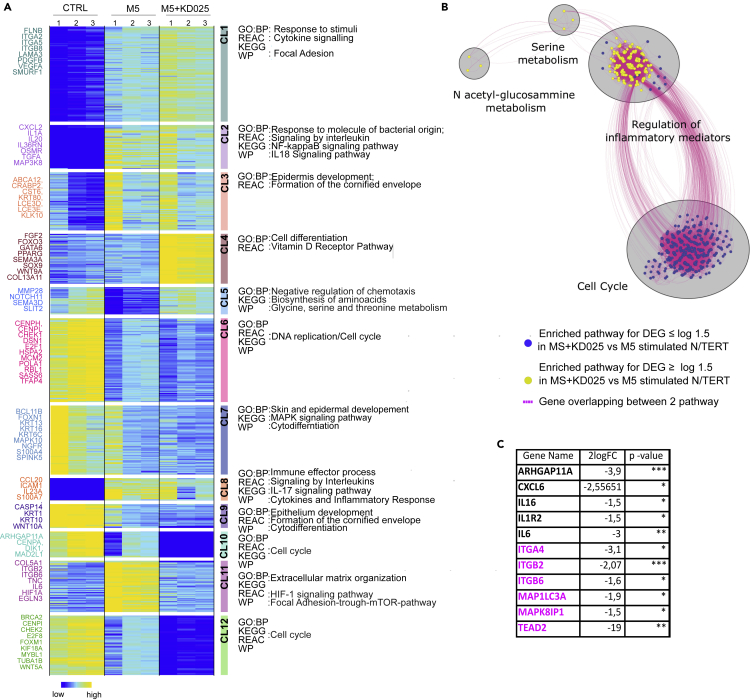
Transcriptomic changes elicited by ROCK2 inhibition in keratinocytes exposed to inflammatory stimuli (A) Heatmap of K-means clustering of variably expressed genes with at least 2 log2 fold change among N/TERT keratinocytes stimulated or not with M5 or both M5 and KD025. Expression is displayed as log2 CPM with dark blue indicating lower value and yellow indicating higher values. Based on the expression similarity genes were grouped into 12 clusters (CL1-12), underlined by colored rectangles. Near each rectangle, the most relevant enriched pathways are indicated. On the left, some of the genes for each cluster are shown. (B) Autoannotated enrichment map shows pathways enriched in DEG in both M5 and KD025 compared to M5 condition. Blue and yellow nodes represent pathways enriched in downregulated and upregulated genes, respectively. Similar pathways with many common genes are connected by dotted pink lines. Groups of similar pathways are annotated with an enclosing gray circle and a summarizing label. (C) Selected hub genes between “regulation of inflammatory mediators” and “cell cycle regulation” relevant for psoriasis or involved in YAP signaling pathway (all the hub genes between the same two network are shown in Figure S6). Note that the pathway names are assigned by a particular database and may vary depending on the database.

To address whether ROCK2 regulates gene expression in keratinocytes exposed to a psoriatic-like cytokine environment, we performed RNA sequencing in N/TERT keratinocyte monolayers treated either with M5 or with a combination of M5 and the ROCK2 inhibitor. As expected, we observed that M5 stimulation substantially affected the keratinocyte molecular signature. The ROCK2 inhibition partially reverted some pathways in the M5-driven transcriptional reprogramming of keratinocytes, whereas others remained unchanged or were further affected (Figure 6A).

To identify the biological processes and molecular pathways regulated by ROCK2 in M5-stimulated keratinocytes, we performed hierarchical and K-cluster analyses of the genes with at least 2log fold change among the conditions. Twelve clusters were identified (Figure 6A), and the enrichment analysis showed that inflammatory and epidermal differentiation molecular pathways were dysregulated in M5-stimulated keratinocytes (clusters 2,5,8,11, Figure 6A), thus recapitulating some key molecular features of psoriatic keratinocytes. Interestingly, ROCK2 inhibition by KD025 partially reverted the M5-driven inflammatory signature (clusters 5 and 11, Figure 6A), suggesting that psoriasis-like inflammation in keratinocytes could be in part mediated by ROCK2. We also observed that ROCK2 inhibition substantially downregulated proliferation pathways and upregulated epidermal differentiation pathways compared with M5-stimulated or control keratinocytes (Figure 6B).

Next, we performed enrichment map analysis of differentially expressed genes (DEGs) in keratinocytes treated with both M5 and KD025 or with M5 alone. Our analysis revealed that ROCK2 inhibition downregulated mainly the cell cycle and proliferation pathways. In addition, we observed a downregulation of multiple (but not all) inflammatory pathways, along with an upregulation of metabolic pathways described as anti-inflammatory. Many DEGs were involved in both proliferation and inflammation pathways, meaning the two hubs were strongly interconnected (Figures 6C and S6). Interestingly, among these genes, we found some involved in the YAP regulation (ITGA4, ITGB2, ITGB6, TEAD).

### The *in vitro* keratinocyte inflammatory phenotypes repeat in psoriasis

**Figure 7:**
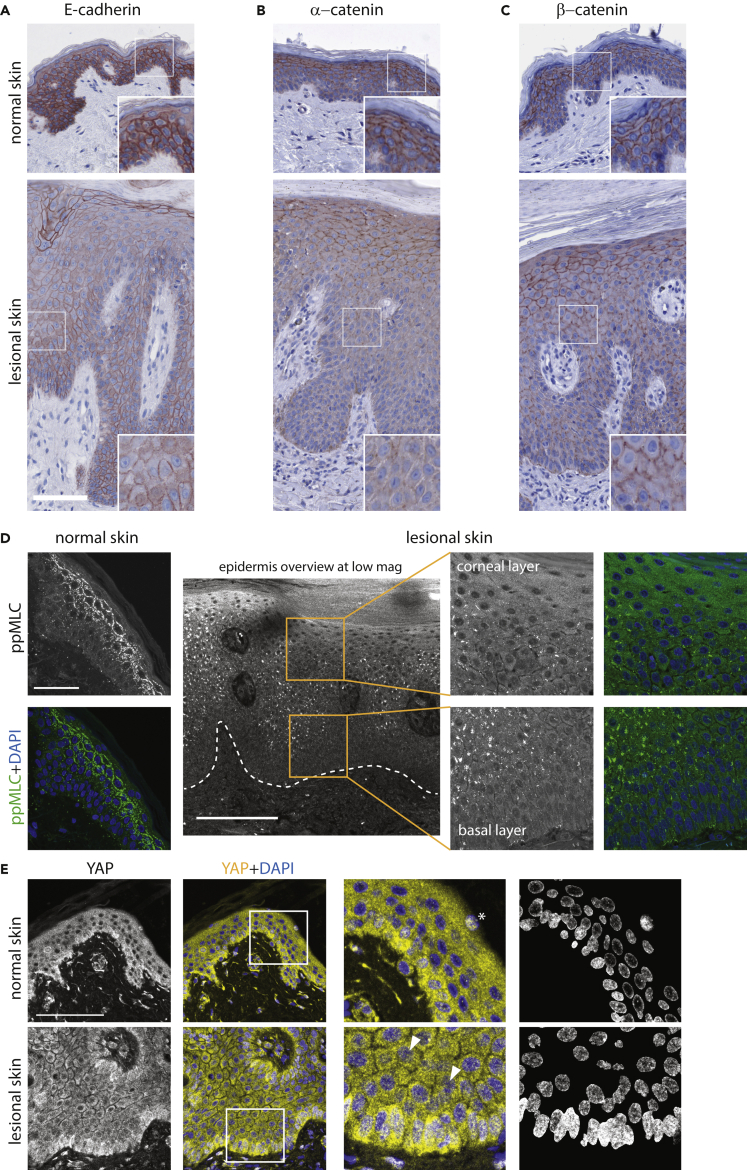
Cell-cell adhesion and mechanotransduction markers in normal human skin and lesional psoriatic skin (A–C) Immunohistochemical staining of E-cadherin (A), α-catenin (B), β-catenin (C). (D) Immunofluorescence staining for ppMLC; dashed line marks the boundary between epidermis and dermis. (E) Immunofluorescence staining for YAP; the last panel shows only thresholded nuclei. Arrowheads show YAP-positive suprabasal nuclei; asterisk shows an example of YAP nucleal signal that is typical for the terminally differentiated dying keratinocytes. Scale bars, 100 μm; 200 μm for the low-mag image in (D) and 50 μm for the inset in (E). Four donors for each group were analyzed.

We have shown that induction of inflammation causes AJ disassembly and altered mechanosignaling in epidermal keratinocytes *in vitro*. Finally, we investigated if these phenotypes were relevant in the context of human disease. We analyzed skin biopsies of psoriatic patients and compared them with healthy donors. Similar to our *in vitro* models, we found that the expression level and AJ localization of E-cadherin, α-catenin, and β-catenin were decreased in lesional psoriatic epidermis (Figures 7A–7C, compare with Figure 1E). Notably, the ppMLC labeling resembled the pattern obtained in RHEs: a strong and continuous labeling at the cell-cell borders in the spinous and granular epidermal layers in the healthy epidermis and decreased or fragmentary labeling in the diseased epidermis (Figure 7D, compare with Figure 2J). Of note, a direct comparison of phosphoprotein levels between different paraffin-embedded biopsies must be done with caution,^80^ and therefore, in our analysis we relied mainly on the ppMLC pattern rather than intensity.

Lastly, an increased nuclear localization of YAP was observed in the keratinocytes above the basal cell layer in the psoriatic epidermis, whereas it was more excluded from the nucleus in suprabasal cells in healthy controls (Figure 7E, compare with Figure 3D). Together, these data indicate that our experimental systems recapitulate several key features of the inflammatory response in the human epidermis in general and, specifically, the signaling responses characteristic for psoriasis.

## Discussion

The integration of cytoskeleton-dependent mechanosensing and mechanotransduction pathways is critical for the regulation of cell and tissue homeostasis in response to inflammation. Here for the first time we describe the cytoskeletal inflammatory phenotype in human keratinocytes and stratified epidermis. We demonstrate that inflammatory cytokines induce activation of the Rho pathway associated with destabilization of epidermal AJs and YAP nuclear translocation, largely through ROCK2 function, which is separate from NMII activation.

### Increased actin-myosin contractility and decreased AJ protein engagement result in the disruption of AJs

The actin-myosin tension has a dual role in regulating epithelial and endothelial cell-cell contact, either stabilizing AJ structure or driving its disassembly,^81^ which also depends on the alignment and dynamics of AJ-associated cytoskeletal structures. For example, the rearrangement of linear perijunctional actomyosin belt into radial stress fibers represents a hallmark of AJ disassembly and reduced barrier function. The tension-induced stabilization of AJs is dependent on vinculin, which is recruited in an NMII-dependent manner and can bind directly to both α- and β-catenin.^25^^,^^27^^,^^82^ Indeed, we found an increased ability for vinculin recruitment to AJs in the cytokine-stimulated keratinocytes. At the same time, in response to the inflammatory cytokines, keratinocytes reorganized continuous AJs into punctate adhesions associated with radial stress fibers.

Another contributing factor for the AJ disorganization and increased permeability under inflammatory conditions is a lower expression and/or higher internalization of AJ proteins and their subsequent degradation. While we detected a decrease in E-cadherin and β-catenin proteins, we did not find a significant decrease in their mRNAs, making the internalization and degradation scenario more likely.

Thus, two different mechanisms are likely to mediate keratinocyte AJ disassembly in response to inflammation: the increased actin-myosin tension accompanied by vinculin recruitment to AJs and the decreased engagement of AJ proteins. Moreover, a dissociation of E-cadherin cell-cell contacts itself may contribute to the Rho-ROCK activation.^83^

### MLC phosphorylation response to inflammation is different in monolayer and RHE cultures

Surprisingly, we observed different MLC phosphorylation response to M5 stimulation in non-differentiated monolayer cultures, where the phosphorylation (especially ppMLC) was upregulated, and in multi-layered RHEs, where it was downregulated.

The mechanical signals from distinct mechanosensitive structures (such as focal adhesions and AJs) can be integrated due to continuity of the cytoskeletal networks.^84^ In monolayer cultures, ppMLC localized to the stress fibers anchored to focal adhesions that keratinocytes develop on a stiff substrate such as coverslip. The activation of Rho induced in response to the cytokine stimulation allowed for increased MLC phosphorylation and contractile force development on a rigid substrate. In line with this, stimulated keratinocytes exhibited enlarged actin stress fibers and focal adhesions indicating elevated basal contractile forces.

In contrast to the cells cultured on coverslips, the keratinocytes in multi-layered RHEs are surrounded by environment consisting of other cells. This could trigger a marked force redistribution from the matrix adhesions (focal adhesions) to intercellular junctions. Indeed, the observed ppMLC pattern in control RHE cultures and skin biopsies corresponded mainly to cell borders. The pattern of continuous ppMLC enrichment around cell borders was largely absent under inflammatory conditions. One possible explanation for this phenomenon is that the cytokine-induced contractile force development cannot be sustained at the weakened cadherin-dependent adhesion sites due to the decrease in AJ protein engagement. Even though the cells of the RHE basal layer still make a contact with the synthetic membrane, but its porous structure may prevent the geometric possibility for the formation of large focal adhesions required for substantial force development. The lack of strong actin anchoring from either type of adhesion structures, in principle, may explain a downregulation of ppMLC and would also cause a barrier “leakage” in the cytokine-stimulated epidermis.

On the other hand, the difference in NMII activation patterns may not be purely mechanical or stiffness dependent but rely on the different differentiation state of the cells in monolayers and the cells in stratified epidermis. Indeed, our experiments with soft substrates showed that M5 stimulation still induced prominent MLC phosphorylation and substantial spreading in keratinocyte monolayers on soft substrates. Moreover, junctional ppMLC enrichment was also observed in the central areas of large cell islands cultured on soft hydrogels, where cells are densely packed and may start differentiating program. Assuming that the MLC phosphorylation at the sites of cell-cell junctions increases with keratinocyte differentiation (indeed, it is stronger in the granular than in basal or suprabasal layer of the epidermis), a partial de-differentiation in M5-stimulated stratified epidermal layers might be responsible for the ppMLC loss.

Overall, our data suggest that the keratinocyte inflammatory response *in vitro* depends on the experimental model and the inflammation-induced mechanoregulation in stratified epidermis may differ from single-layered simple epithelia or endothelium.

### YAP in keratinocytes is regulated via AJ integrity

Rho signaling is universally recognized as a positive regulator of YAP/TAZ,^31^^,^^67^ presumably due to the effects on the NMII- and cofilin-dependent reorganizations of the focal adhesion-associated actin cytoskeleton that initiate mechanotransduction pathways. Indeed, studies showed the importance of integrin signaling for the YAP function in epithelial cells.^79^^,^^85^ Moreover, a pharmacological pan-ROCK or Src inhibition promoted YAP nuclear exclusion in the keratinocyte cell line HaCaT.^79^

However, the effects of Rho/ROCK pathway inhibition on YAP in keratinocytes are disputable. In a number of studies, pan-ROCK inhibitor Y-27632 has been shown to promote YAP nuclear entry, proliferation, and migration in primary human keratinocyte monolayers and skin explant cultures.^41^^,^^86^^,^^87^^,^^88^ In our experiments, treatment of human keratinocytes with Y-27632 caused a loss of MLC phosphorylation and AJ disassembly and increased YAP nuclear translocation in both control and cytokine-stimulated cells. Similarly, this AJ and YAP phenotype was induced by blebbistatin treatment. Our data are consistent with the study of Walko and colleagues,^41^ which proposed the contact inhibition being a predominant factor for YAP regulation in human keratinocyte colonies. Thus, the cellular response might be governed by the balance between two major ways of YAP regulation: a positive regulation via substrate adhesion and a negative regulation via cell-cell adhesion. The discrepancy in Y-27632 effects might, therefore, be cell type dependent. The regulation via cadherin-dependent adhesion could be a predominant factor for primary/normal keratinocytes, whereas the substrate attachment will be dominating for transformed keratinocytes such as HaCaT or for mesenchymal cells. Indeed, we observed that YAP nuclear targeting in normal N/TERT keratinocytes in response to M5 stimulation was not strongly dependent on the substrate stiffness.

In our experiments, the inflammation-induced reorganization of AJs and Rho-dependent strengthening of substrate adhesions both could play a promoting role for the YAP nuclear targeting. The addition of Y-27632 to the M5-stimulated cells resulted in the utmost disruption of AJs leading to even stronger YAP nuclear entry. In parallel, the level of β-catenin engagement at AJs decreased but did not result in the β-catenin nuclear targeting in any of the experimental conditions, likely because of the inactive Wnt signaling.

### ROCK2 but not NMII activation is central for AJ and YAP regulation in keratinocyte inflammatory responses

It has been proposed that the role of the Rho/ROCK pathway in regulating AJs is the stimulation of NMII contractility. Our experiments showed that YAP nuclear translocation does not correspond to the level of MLC phosphorylation in keratinocytes; however, it strongly correlates with the integrity of AJs. We showed that the inhibition of ROCK2 in the cytokine-treated keratinocytes stabilized AJs, which is consistent with various studies that reported a role of ROCK2 in destabilization of cell-cell adhesion^89^ and in promoting epidermal proliferation in mouse models.^73^^,^^90^

The rapid effect of KD025 on AJs and, therefore, YAP nuclear exclusion, as observed in our experiment with blebbistatin, might be explained through modulation of the AJ-associated cytoskeleton, e.g., by negative regulation of cofilin through its phosphorylation^91^ that affects actin turnover at the AJs and is crucial for their integrity.^92^ At the same time, it appeared to be NMII contractility independent, first, because the tension-dependent vinculin recruitment to AJs was well retained in KD025-treated M5-stimulated cells. Secondly, the inhibition of the NMII motor by blebbistatin and simultaneous inhibition of ROCK2 resulted in cytoplasmic YAP localization, whereas nuclear YAP was promoted by blebbistatin alone.

Beyond the cytoplasmic functions, ROCK2 (but not ROCK1) has been shown to localize to the nucleus and takes part in gene regulation in different cell types.^93^^,^^94^^,^^95^^,^^96^ Our RNA sequencing data reveal that many genes are differentially expressed in M5-stimulated keratinocytes when ROCK2 was inhibited. The majority of these genes were associated with cell cycle and inflammatory, but not cell-cell adhesion, pathways. Thus, we report both transcriptional and non-transcriptional effects of ROCK2 inhibition in the regulation of keratinocyte inflammatory response.

In the context of inflammation, ROCK2 inhibitor KD025 previously has been shown to suppress the production of IL17A, IL-10, and IL-21 by human T-cells via the STAT3-dependent mechanism,^76^^,^^97^ and a phase-2 clinical trial for its application for psoriasis vulgaris has been recently completed (clinicaltrials.gov NCT02317627). Here, we present the data indicating that KD025 has another function principle, namely, acting directly on human epidermal keratinocytes regulating their adhesion, barrier function, and mechanosignaling. Therefore, it may be considered to be tested as a topical treatment in skin inflammation.

### Conclusions

**Figure 8:**
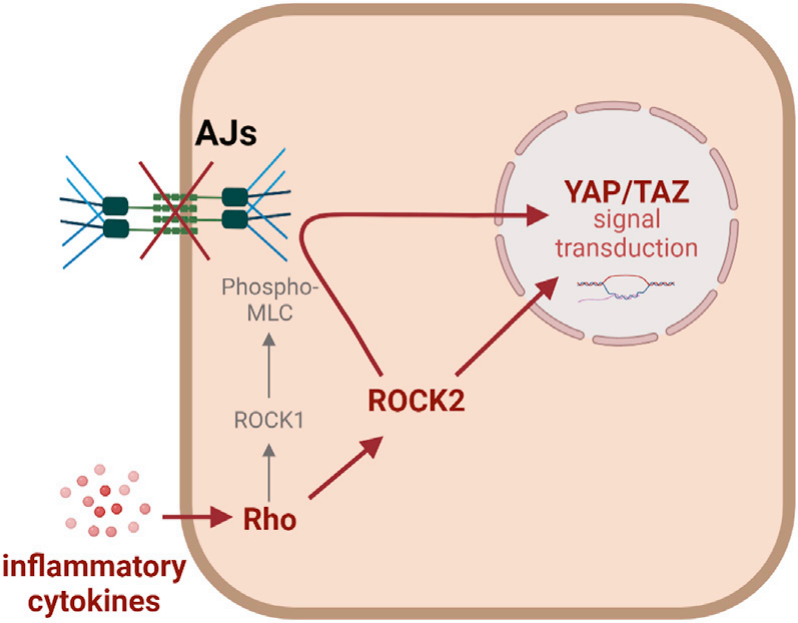
A model for ROCK2-dependent regulation of keratinocyte mechanoresponse to inflammation Figure was created with BioRender.com.

To summarize, we show that psoriasis-like inflammation debilitates AJs and upregulates mechanosignaling in human epidermis. These effects partially rely on the activity of ROCK2 but are not mediated via the increase in MLC phosphorylation (Figure 8). Pharmacological inhibition of ROCK2 is able to partially rescue some aspects of the cytokine-induced inflammatory phenotypes in keratinocytes by restoring continuous AJs and intercellular permeability, inhibiting YAP translocation to the nucleus and regulating transcription of inflammation-relevant genes. ROCK2 inhibition, therefore, may be a promising strategy for the topical treatment of cutaneous inflammation in general and psoriasis in particular.

### Limitations of the study

The experiments were performed using normal human keratinocytes and produced from them RHE models, which give results much more relevant to human pathology than transformed cell lines or animal models. At the same time, the study does not consider how inflammatory stimuli and ROCK2 inhibition affect the interactions between epidermis, dermis, and immune system. M5 cytokine cocktail is a well-characterized and widely used tool to model psoriasis-like inflammation *in vitro*; however, it does not mimic all the features of real psoriatic epidermis, such as keratinocyte hyperproliferation. Even though KD025 is a well-characterized ROCK2 inhibitor already used in numerous studies and clinical trials, we cannot exclude some non-specific effects.

## STAR★Methods

### Key resources table

**Table:** 

REAGENT or RESOURCE	SOURCE	IDENTIFIER
**Antibodies**		

E-cadherin	Cell Signaling Technology	Cat#14472 RRID:AB_2728770
E-cadherin	Abcam	Cat#ab40772 RRID:AB_731493
Phospho-cofilin (Ser3)	Cell Signaling Technology	Cat#3313 RRID:AB_2080597
Phospho Myosin Light Chain 2 (Ser19)	Cell Signaling Technology	Cat#3671 RRID:AB_330248
phospho-YAP (Ser127)	Cell Signaling Technology	Cat#4911 RRID:AB_2218913
ROCK1	Abcam	Cat#ab97592 RRID:AB_10688425
ROCK2	Abcam	Cat#ab71598 RRID:AB_1566688
TAZ (WWTR1)	Sigma-Aldrich	Cat#HPA007415 RRID:AB_1080602
Vincilin	Sigma-Aldrich	Cat#V9131 RRID:AB_477629
YAP	Santa Cruz Biotechnology	Cat# sc-101199 RRID:AB_1131430
YAP	Proteintech	Cat#13584-1-AP RRID:AB_2218915
α-catenin	ThermoFisher Scientific	Cat#13-9700 RRID:AB_2533044
β-catenin	Abcam	Cat#ab32572 RRID:AB_725966

**Biological samples**		

Skin biopsies (psoriasis patients)	Department of Dermatology of the Geneva University Hospitals	N/A
Skin biopsies (healthy donors)	Department of Plastic and Reconstructive Surgery and Division of Clinical Pathology of the Geneva University Hospitals	N/A

**Chemicals, peptides, and recombinant proteins**		

IL-17A	R&D Systems	Cat#317-ILB
IL-1α	R&D Systems	Cat#200-LA
Oncostatin M	R&D Systems	Cat#295-OM
IL-22	MACS Miltenyl Biotec	Cat#130-096-295
TNFα	PeproTech	Cat#300-01A
Blebbistatin	EMD Millipore	Cat#203391
KD025	MedChem Express	Cat# HY-15307
Y-27632	Abcam	Cat#ab120129
Biotin	Tocris	Cat#7302

**Critical commercial assays**		

Active Rho Pull-Down and Detection kit	Thermo Scientific	Cat#16116
Vascular permeability assay kit	Sigma-Aldrich	Cat#17-10398
Substrates of 0.5 kPa stiffness (Matrigen easy coat hydrogels), 35 mm dishes	Cell Guidance systems	Cat# PS35-EC-0.5-EA
Substrates of 12 kPa stiffness (Matrigen easy coat hydrogels), 35 mm dishes	Cell Guidance systems	Cat# PS35-EC-12-EA
Substrates of 50 kPa stiffness (Matrigen easy coat hydrogels), 35 mm dishes	Cell Guidance systems	Cat# PS35-EC-50-EA

**Deposited data**		

RNA-sequencing data	NCBI Gene Expression Omnibus depository	GSE202522
Other original data	Mendeley	https://doi.org/10.17632/psbgw2vcrg.1

**Experimental models: Cell lines**		

N/TERT immortalized normal human keratinocytes	Prof. E.H. van den Bogaard, Radboud University Medical Center, Nijmegen, The Netherlands	N/A
Primary human keratinocytes from foreskin	Polish-American Children’s Hospital, Krakow, Poland	N/A

**Oligonucleotides**		

GAPDH forward primer 5′-TCGGAGTCAACGGATTTGGT-3′	Microsynth (Schützenstrasse, Balgach, Switzerland)	N/A
GAPDH reverse primer 5′-TGAAGGGGTCATTGATGGCA-3′	Microsynth	N/A
IL-8 forward primer 5′-GCTCTCTTGGCAGCCTTCCT-3′	Microsynth	N/A
IL-8 reverse primer 5′-TTAGCACTCCTTGGCAAAACTG-3′	Microsynth	N/A
CCL20 forward primer 5′-GCT GTG ACA TCA ATG CTA TCA TCT T-3′	Microsynth	N/A
CCL20 reverse primer 5′-CGC ACA CAG ACA ACT TTT TCT TTG-3′	Microsynth	N/A
IL-36γ forward primer 5′-GCA CTC CAG GAG ACG CTG AT-3′	Microsynth	N/A
IL-36γ reverse primer 5′-GGT CCA CAC TTG CTG ATT CAA A-3′	Microsynth	N/A
E-cadherin forward primer 5′-AGG CCA AGC AGC AGT ACA TT-3′	Microsynth	N/A
E-cadherin reverse primer 5′-CAT TCA CAT CCA GCA CAT CC-3′	Microsynth	N/A
α-catenin forward primer 5′-TCC TGC TGT GTC ATG GAA-3′	Microsynth	N/A
α-catenin reverse primer 5′-GCT TTG AAC TCG CTG AGG-3′	Microsynth	N/A
β-catenin forward primer 5′-TCT GAG GAC AAG CCA CAA GAT TAC A-3′	Microsynth	N/A
β-catenin reverse primer 5′-TGG GCA CCA ATA TCA AGT CCA A-3′	Microsynth	N/A

**Software and algorithms**		

ImageJ	Schneider et al., 2012^98^	https://imagej.nih.gov/ij/
GraphPad Prism 8	GraphPad	https://www.graphpad.com/scientific-software/prism/
R/Bioconductor package edgeR v. 3.18.1.	Robinson et al., 2010^99^	R/Bioconductor package edgeR v. 3.18.1.
Cluster 3.0	de Hoon et al., 2004^100^	https://www.encodeproject.org/software/cluster/
gProfiler	Raudvere et al., 2019^101^	https://biit.cs.ut.ee/gprofiler/gost
PathMe viewer	Domingo-Fernandez et al., 2019^102^	https://pathme.scai.fraunhofer.de/
Cytoscape	Shannon et al., 2003^103^	https://cytoscape.org/

**Other**		

KBM-Gold Keratinocyte Growth Medium BulletKit	Lonza	Cat#00192060
Keratinocyte-SFM Medium (Kit) with L-glutamine, EGF, and BPE	Gibco	Cat#17005075
CnT-Prime Epithelial Culture Medium	CellnTec	Cat#CnT-PR
CnT-Prime 3D Barrier Culture Medium	CellnTec	Cat# CnT-PR-3D
Thincert chambers for RHE models	Greiner Bio-One	Cat# 665640

### Resource availability

#### Lead contact

Further information and requests for resources and reagents should be directed to and will be fulfilled by the lead contact, Maria Shutova (maria.shutova@unige.ch).

#### Materials availability

This study did not generate new unique reagents.

### Experimental model and subject details

#### Human skin samples

Psoriatic skin biopsies were taken from untreated adult male and female patients presenting at the Department of Dermatology of the Geneva University Hospitals in Switzerland (age 38–50). Healthy skin biopsies were taken from adult female patients presenting at the Department of Plastic and Reconstructive Surgery and Division of Clinical Pathology of the Geneva University Hospitals in Switzerland (age 34–58). The foreskin biopsies for the isolation of primary keratinocytes were taken from male children (age 1–16) undergoing surgery at the Polish-American Children’s Hospital, Krakow, Poland. This study was conducted according to the Declaration of Helsinki and approved by the local ethical committee of the University Hospitals of Geneva, Switzerland, and the Jagiellonian University according to Polish law (No. 1072.6120.9.2017). Written informed consent was obtained for each individual.

#### Monolayer cell culture

Primary human keratinocytes were isolated from juvenile foreskin biopsies after enzymatic digestion with 10 U/mL dispase (Gibco) and with 0.05% trypsin, 2 mmol/L EDTA subsequently (Sigma-Aldrich). Cells were cultured in serum free Gold Keratinocyte Growth Medium (Lonza, Switzerland) in a humidified atmosphere with 5% CO_2_ at 37°C and used for the experiments at passage 3–4.

The human N/TERT male keratinocyte cell line (N/TERT-1) was obtained from Prof. Ellen H. van den Bogaard Laboratory (Radboud University Medical Center, Nijmegen, The Netherlands) thanks to the courtesy of the J. Rheinwald laboratory (Harvard Medical School, Boston, USA). N/TERT cells were cultured in keratinocyte-serum free medium (K-SFM, Gibco), supplemented with 25 μg/mL bovine pituitary extract (BPE) (Invitrogen), 0.2 ng/mL EGF (Invitrogen), 300 μM CaCl_2_ (Sigma-Aldrich) and antibiotics in a humidified atmosphere with 5% CO_2_ at 37°C and passaged at 30–40% confluency. For the experiments, N/TERT cells were switched to the experiment medium composed of 50% K-SFM and 50% DF-K medium. DF-K was prepared as follows: DMEM:Ham’s F12 1:1, supplemented with 25 μg/mL bovine pituitary extract (BPE) (Invitrogen), 2 mM L-glutamine (Invitrogen), 0.2 ng/mL EGF (Invitrogen) and 300 μM CaCl_2_ (Sigma-Aldrich). Stimulation with M5 cytokines and inhibitors was performed at the time of medium switch and lasted 24 or 48 h. The original K-SFM medium was kept for the short-term experiments with blebbistatin.

Notably, the concentration of calcium was maintained lower for the primary keratinocytes compared with N/TERT, because higher calcium drove the primary cells to differentiation and restricted their growth. Due to this lower calcium, primary cells formed loser cell-cell contacts than N/TERT in our experimental settings.

For the experiments with the substrates of variable stiffness, N/TERT were plated at the density of 100′000 cells on Matrigen Petrisoft 35 mm Dish Easy Coat of 0.5, 12 and 50 kPa hydrogels (Cell Guidance Systems) coated with human collagen type IV (Sigma-Aldrich) at 40 μg/mL in 0.02M acetic acid for 2 h at 37C. Cells were cultured for two days before the cytokine stimulation.

#### Reconstructed human epidermis (RHE) model

RHEs were generated using N/TERT keratinocytes and primary juvenile keratinocytes at passage 3.^78^ The cells were plated at 250′000 in 0.5 mL CnT PRIME medium (CELLnTEC) in the thincert chambers (Greiner Bio-One), placed into 12 well plate, with the wells containing 1 mL of CnT PRIME medium outside the chamber. Next day, the medium inside the chamber was refreshed to remove not attached cells. After two more days, the medium was replaced with CnT PRIME 3D barrier medium (CELLnTEC) in both inside and outside chamber. After 24 h, the thinserts were transferred into deep well plates (Greiner, Cat# 665110) with 4 mL CnT PRIME 3D barrier medium in the bottom, whereas the liquid above the cell layer was dried (counted as Day 0 of airlift). The medium was refreshed on Days 3, 5 and 7. At Day 10 of culturing at the air-liquid interface, RHEs were stimulated with M5 cytokines for 24 or 48 h.

### Method details

#### M5 cytokine stimulation

The following cytokines were used at 10 ng/mL as M5 cocktail: IL-17A, IL-1α, Oncostatin M (all from R&D Systems), IL-22 (MACS Miltenyl Biotec), TNFα (PeproTech London, UK).

#### Small molecule inhibitors

The following inhibitors were used: 20 μM Y-27632 (Abcam), 20 μM KD025 (MedChem Express), 50 μM (−)-Blebbistatin (Calbiochem). The inhibitors were diluted in DMSO, with the final DMSO concentration of 0.2% (0.5% for the blebbistatin experiments) in the culture medium. All controls in the experiments involving inhibitors were treated with DMSO at the corresponding concentration. The inhibitors were added simultaneously with the cytokine stimulation.

#### Permeability assays

For the permeability assay of keratinocyte monolayers, we used an *in vitro* vascular permeability assay kit (Sigma-Aldrich). The N/TERT were plated on the 18 mm coverslips prepared according to the manufacturer’s protocol and the experimental treatment was performed as described above. Then, the samples were treated with Fluorescein-Streptavidin for 5 min and fixed with 4% paraformaldehyde for 15 min, washed in PBS 3 times, mounted on slides and imaged with the confocal microscope. The areas inside large keratinocyte islands were chosen for the imaging.

To test the basal-to-apical permeability of RHE cultures, we adapted a method used in.^104^ The RHEs were cultured and stimulated as described above. After 24 h of stimulation, the biotin stock solution (10 mg/mL in water, Tocris, Cat.# 7302) was added directly to the media at the bottom of the inserts to the final concentration of 0.5 mg/mL. The cultures were incubated for 1 h. Then RHEs were fixed in 4% formaldehyde overnight and embedded in paraffin. 5 μm sections were cut, deparaffinized and directly stained with Fluorescein-Streptavidin (Sigma-Aldrich, Cat.# 17–10398) at 1:2000 30 min in PBS.

For the apical-to-basal permeability assay on RHE cultures, RHE cultures were moved from deep well plates to standard 12 well plates after 11 days of airlift (10 days of standard airlifted culture followed by 24 h of stimulation). In 12 well plate, 1.850 mL medium with continuous respective stimulation was added to the bottom well and 0.6 mL medium with continuous respective stimulation and 0.2 mM Na-Fluorescein (Sigma-Aldrich) was placed in the upper chamber, on top of the cornified layer. The fluorescence intensity in the bottom well was measured after 24 h of incubation with fluorescein (meas. filter 492 nm, ref. filter 570 nm).

#### Immunofluorescence staining and imaging of cells

Coverslips with cells were fixed with 4% paraformaldehyde in PBS at room temperature for 15 min and permeabilized with 0.1% Triton X-100 (Sigma-Aldrich) in PBS for 5 min. Cells were stained for 1 h with primary antibodies followed by secondary antibodies: Alexa Fluor-568-conjugated donkey anti-mouse IgG (Invitrogen), Alexa Fluor-488-conjugated donkey anti-rabbit IgG secondary antibodies (Invitrogen), and Alexa Fluor 488 or -647 phalloidin. The coverslips were mounted on the slides using DAPI Fluoromount-G (SouthernBiotech). z stack confocal images were acquired using the LSM800 Airyscan confocal microscope (Zeiss) at 40x or 63x magnification.

#### Immunostaining and imaging of histological samples

Skin tissue samples or RHEs were fixed in 4% formaldehyde overnight, embedded in paraffin, and 5 μm sections were cut and deparaffinized. Antigen retrieval was performed in 10 mM/L citrate buffer pH 6.0 for 30 min at 95°C.

For the immunohistochemical staining (IHC), the activity of endogenous peroxidase was quenched with Bloxall blocking solution (Vector Laboratories). Sections were stained using the R.T.U. Vectastain Kit with the ImmPACT AMEC Red Substrate Kit (Vector Laboratories) according to the manufacturer’s protocol. Sections were counterstained with haematoxylin solution, mounted with Glycerol Mounting Medium (Dako, Denmark) and then brightfield images were taken at 20x magnification using Zeiss Axioscan.Z1 (Zeiss, Germany).

For immunofluorescence, sections were blocked with 3% BSA and 5% normal goat serum in PBS for 1 h. Next, sections were incubated for 2 h with primary antibodies followed by 1 h incubation with Alexa Fluor 488-conjugated donkey anti-rabbit and Alexa Fluor 555-conjugated donkey anti-mouse IgG secondary antibodies (Invitrogen), all in PBS with 1% BSA (Sigma-Aldrich) and 0.1% Tween 20 (Sigma-Aldrich), and mounted with DAPI Fluoromount-G (SouthernBiotech). z stack confocal images of fluorescent samples were acquired using the LSM800 Airyscan confocal microscope (Zeiss) at 40x magnification.

#### Active Rho assay

N/TERT cells were plated on 100 mm Petri dishes (Corning) at the density of 500′000 cells per dish, and after two days in culture stimulated with M5 in KSFM + DFK (1:1) medium for 24 h. The cells were lysed and the pulldown assay was performed using Active Rho Pull-Down and Detection Kit (Thermo Scientific) according to the manufacturer’s protocol. Then samples were analyzed by Western blotting.

#### Western blotting

Cells were washed in ice-cold PBS with Ca^2^+/Mg^2^+ and lysed in ice-cold RIPA lysis buffer containing Phosphatase Inhibitor Cocktails 2 and 3 and Protease Inhibitor Cocktail (Sigma-Aldrich). After centrifugation at 13′000 g for 10 min at 4°C, supernatants were collected and protein concentration was measured using Bradford assay kit (Bio-Rad) or BCA assay (Fisher Scientific) for Rho-pulldown samples. The samples were mixed with NuPAGE LDS sample buffer and reducing agent (Invitrogen) and heated at 70°C for 10 min 10 μg total protein was loaded onto a 4–12% NuPAGE BT gradient gel (Invitrogen), resolved by SDS-PAGE, and transferred to a 0.45 μm polyvinylidene fluoride membrane in NuPAGE Transfer Buffer at 20V for 1h using a Trans-Blot SD Semi-Dry Transfer Cell (Bio-Rad). The membrane was blocked for 30 min with 5% BSA in PBS buffer containing 0.1% Tween 20 (Sigma-Aldrich), incubated with primary antibodies in PBS/Tween 20 overnight at 4°C, washed in PBS/Tween 20, incubated with appropriate HRP-conjugated secondary antibodies for 1 h, and developed with Super-Signal West Pico PLUS Chemiluminescent Substrate (Thermo Fisher Scientific).

#### RNA extraction and RT-qPCR

Total RNA was isolated using the Quick-RNA MiniPrep or Micro-Prep kit (Zymo Research Corporation), according to the manufacturer’s protocol. cDNA was synthesized from 1 μg of total RNA using the Super-Script II Reverse Transcriptase (Thermo Fisher Scientific). Genes of interest were quantified with PowerUp SYBR green Master Mix (Bio-Rad Laboratories AG, Switzerland) on StepOne Plus instrument (Applied Biosystems) and normalized to GAPDH mRNA or 18S rRNA using a comparative method (2-ΔCt). Non-reverse-transcribed RNA samples and buffer were included as negative controls. The primers are listed in the key resources table.

#### RNA sequencing

RNA was isolated from N/TERT cells treated with 0.2% DMSO (control), M5 + 0.2% DMSO, and M5 + 20uM KD025 for 24 h in three independent experiments with Qiagen RNAeasy microkit. Integrity and quantity of RNA were assessed with Bioanalyzer (Agilent Technologies) (RIN ranging from 7.5 to 10). cDNA libraries were constructed using the Illumina TruSeq RNA stranded Kit according to the manufacturer’s protocol starting from poly-A RNA. Pools of 18 libraries were loaded at 2 nM for clustering on two lanes of a Single-read Illumina Flow cell. Reads of 50 bases were generated using the TruSeq SBS chemistry on an Illumina HiSeq 4000 sequencer. FastQ reads were mapped to the ENSEMBL reference genome (GRCh38.89) using STAR, version v.2.5.3a, with standard settings. The transcriptome metrics were evaluated with the Picard tools v.1.141. The table of counts with the number of reads mapping to each gene feature of the UCSC human hg38 reference was prepared with HTSeq v0.9.1. The differential expression analysis was performed using R/Bioconductor package edgeR v. 3.18.1.^99^ Briefly, the counts were normalized according to the library size and filtered. The genes were filtered on expression levels. As required by the experimental design, paired t-test was used to assess the differentially expressed genes. The differentially expressed gene p values were corrected for multiple testing errors with a 5% FDR (false discovery rate) according to the correction by Benjamini-Hochberg (BH). The library preparation, sequencing, and read mapping to the reference genome were performed by the Genomics Plat-form at the University of Geneva, Switzerland.

Hierarchical and k-mean clustering were performed by Cluster 3.0 software.^100^ CPM values were first log transformed then filtered to have at least 2log2 fold change between highest and lowest samples, then we performed hierarchical analysis for both genes and conditions using “average linkage” method, subsequently we organized genes according k-Means cluster analysis for genes. We arbitrary choose a maximum number of 12 cluster. Finally, we used TreeView 3.0 (JAVA) to visualise the k-clustering results.

Enrichment analysis for each cluster was performed by gProfiler (ELIXIR).^101^ Biological process GO (gene ontology), KEGG (Kyoto Encyclopedia of Genes and Genomes), RACTOME and WikiPathways were queried. Results were then analyzed and harmonized using PathMe viewer (Fraunhofer SCAI Department of Bioinformatics) and ROVIGO (Ruđer Bošković Institute) to reduce redundancies between different annotations and databases.^102^^,^^105^^,^^106^

Differentially expressed genes between keratinocytes stimulated with both M5 and KD025 or M5 alone were subjected to over-representation analysis by querying GO biological process, KEGG, REACTOME and WikiPathways. Enrichment map analysis for the significant enriched pathways was the performed and visualised using Enrichment Map Cystoscspe and Autoannotate apps from Cytoscape (Cytoscape consortium).^103^ Networks of interaction for the hubs gens between “regulation of inflammatory mediators” and “ cell cycle” pathway network was built and visualized using STRING v 11.5 (STRING consortium).^107^

### Quantification and statistical analysis

All image analyses were performed using ImageJ software.^98^ Statistical analyses were performed using GraphPad Prism 8 software and appear in the Figure legends.

#### Quantification of vinculin enrichment at AJs

The images for the quantification were taken at 63x and 1.5 zoom, 3–5 fields of view in the middle of an island per experimental condition. For each condition, 8–10 areas of cell-cell contacts were selected in the confocal plane of a particular AJ from the brightest confocal slice in the E-cadherin channel. The AJ areas in the E-cadherin channel were thresholded at identical settings after the subtraction of local E-cadherin background. The integrated intensity of vinculin immunofluorescence was measured in the obtained ROIs. To calculate the mean vinculin intensity for each condition, the integrated vinculin intensity (before or after local background subtraction in the vinculin channel) from all ROIs was divided by the total area of the ROIs in this condition.

#### Quantification of pMLC and ppMLC fluorescence intensity

For the monolayer cultures, confocal images were presented as maximum projections and the total intensities of individual cells were measured after background subtraction. For the RHE cultures, confocal images were presented as maximum projections and the mean intensities from different fields of view (covering all RHE layers) were measured after background subtraction.

#### Analysis of nucleus-to-cytoplasm ratio for YAP/TAZ

For the monolayer cultures, confocal images obtained at 40x magnification (at 20×1.5 for the experiments on soft substrates) were presented as maximum projections, and the mean intensities of ROIs (round ∼20 μm^2^ regions) in the nucleus and in the adjacent cytoplasm were obtained from interphase cells after background subtraction using ImageJ software. For the RHE cultures, confocal images obtained at 40x magnification were presented as the brightest confocal plane, and the mean intensities of ROIs (round ∼10 μm^2^ regions) in the nucleus and in the adjacent cytoplasm were obtained from the cells in the bottom half of the multi-layered epidermis. For the biopsy samples, confocal images obtained at 20x magnification.

#### Permeability assay in monolayer cultures

To estimate the size of the gaps between cells, the maximum projections of FITC-streptavidin confocal images taken at 20x were used. The images were processed in ImageJ as follows: Process - Filters - Gaussian blur (sigma radius 2.00); Image – Adjust – Threshold (maximum possible before it starts selecting areas inside cells); Process – Binary – Make binary. The areas of gaps between cells in the binary images were measured in ImageJ and represented as a percentage of the total area of the images (the areas were summed from 3 to 5 fields of view per coverslip).

#### Biotin permeability assay in RHEs

Confocal images of FITC-streptavidin-stained RHE sections were represented as sum slices and the mean intensity was measured from several fields of view for each section, after background subtraction using ImageJ software.

#### Quantification of band intensity in Western blotting

The black-and-white digital images of the developed Western blots were presented in inverted contrast and 16bit. The local background near the bands of interest was subtracted, the total band intensity was measured and then normalized to the total intensity of the corresponding loading control band.

## Data Availability

The RNA-sequencing datasets related to this article can be found at NCBI Gene Expression Omnibus depository under the access number GSE202522, as listed in the key resources table. Original images and data used for quantification have been deposited at Mendeley and are publicly available as of the date of publication. DOI is listed in the key resources table. Any additional information required to re-analyze the data reported in this paper is available from the lead contact upon request. This paper does not report original code.
