# Supplementary material for: Inflammation modulates intercellular adhesion and mechanotransduction in human epidermis via ROCK2
Source: iScience. 2023 Feb 14;26(3):106195. doi: 10.1016/j.isci.2023.106195 (PMC9986521; doi:10.1016/j.isci.2023.106195)
Supplement: Document S1. Figures S1–S6 [file mmc1.pdf]

iScience, Volume *26*

Supplemental information

Inflammation modulates intercellular adhesion and mechanotransduction in human epidermis via ROCK2

Maria S. Shutova, Julia Borowczyk, Barbara Russo, Sihem Sellami, Justyna Drukala, Michal Wolnicki, Nicolo C. Brembilla, Gurkan Kaya, Andrei I. Ivanov, and Wolf-Henning Boehncke

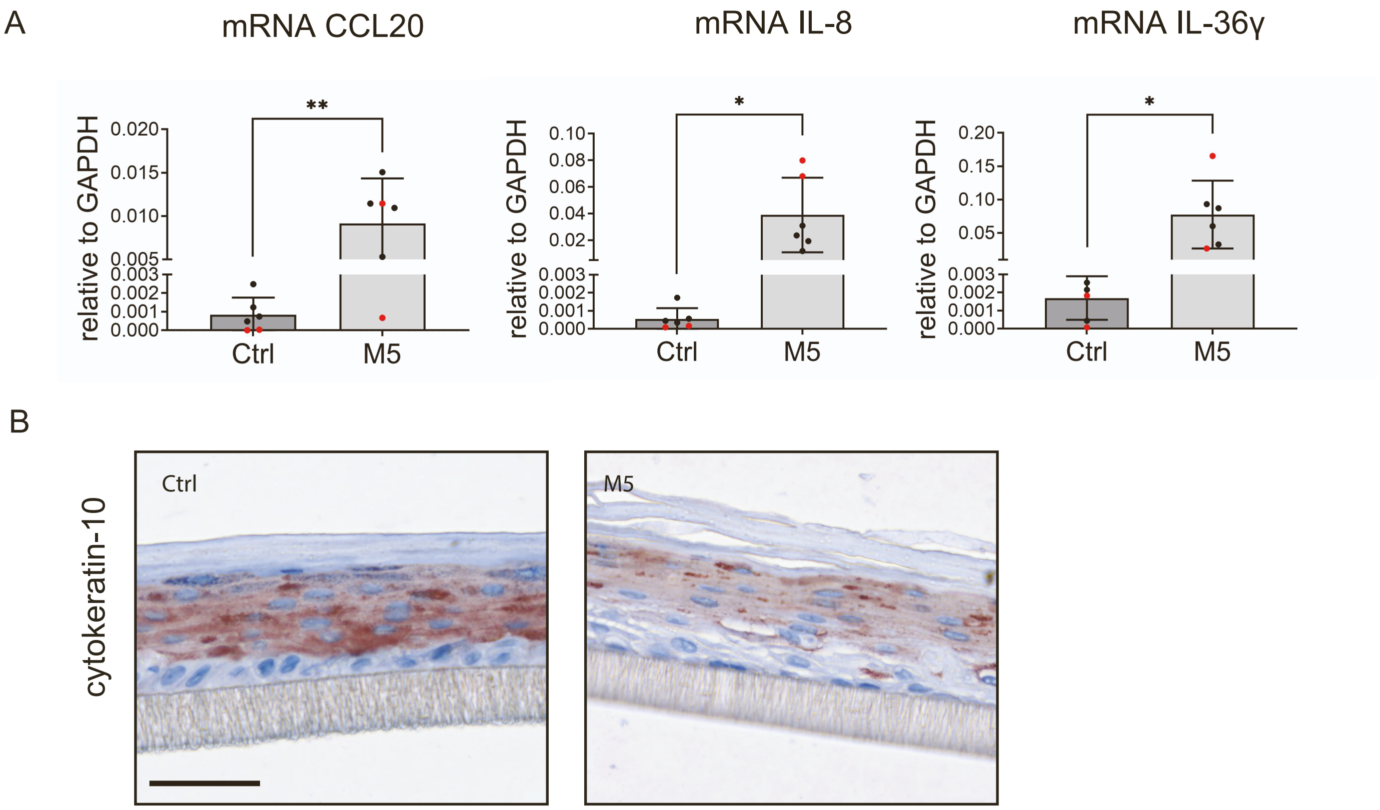

Figure S1. Related to Figure 1.

M5 stimulation causes an inflammatory response in keratinocytes.

(A) Cytokine and chemokine mRNA production in non-differentiated keratinocytes after M5 stimulation for 24 hours (red dots, N/TERT from two independent experiments; black dots, primary keratinocytes from four donors). Mean ± SD. Paired t-test, * $p < 0.05$, ** $p < 0.01$.

(B) Immunohistochemical staining for cytokeratin-10 in RHE models from primary keratinocytes after M5 stimulation for 48 hours. Scale bar, 50 μm.

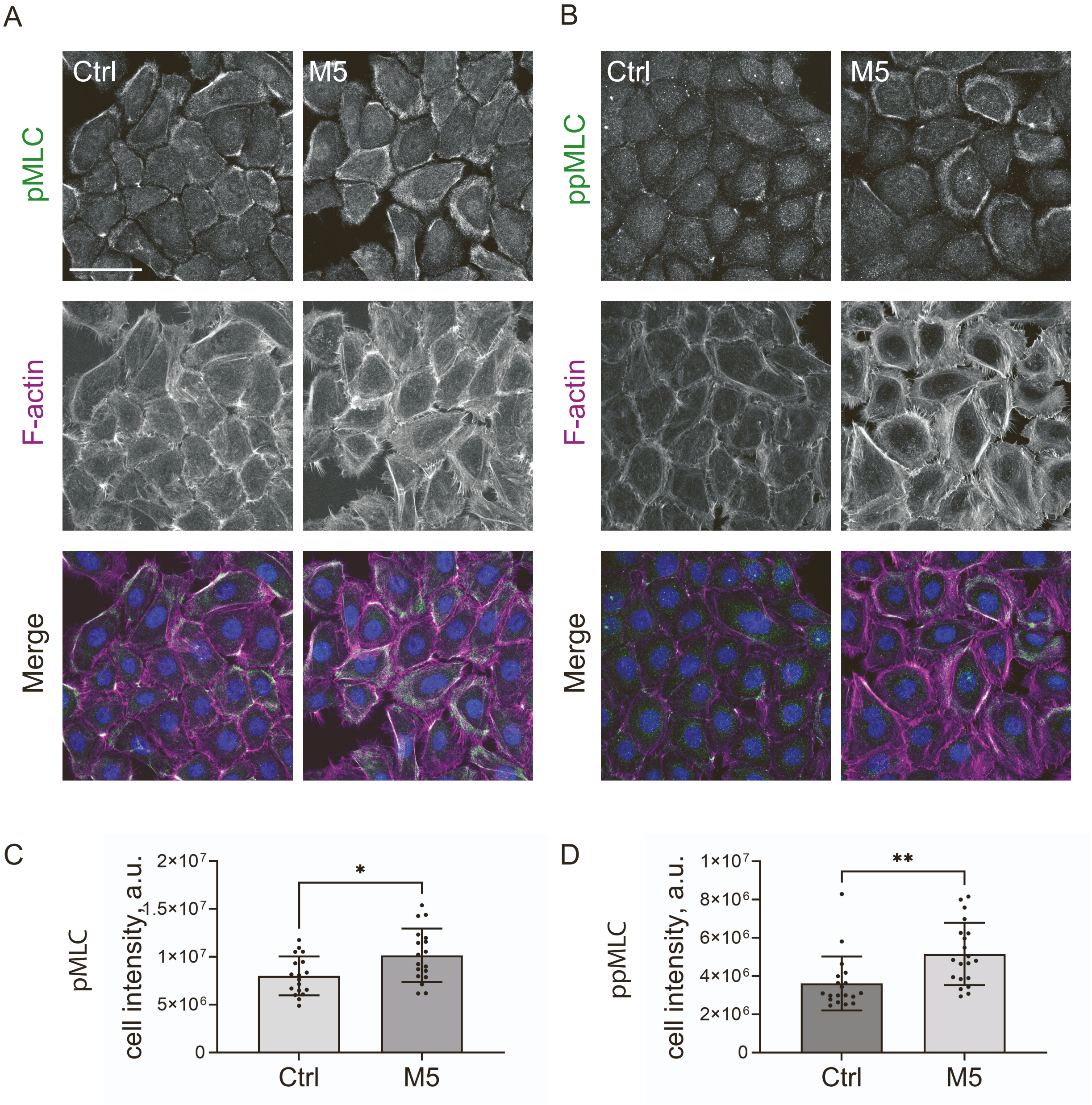

Figure S2. Related to Figure 2.

MLC phosphorylation in non-differentiated primary keratinocytes after 24 hours of M5 stimulation.

(A,B) Immunofluorescence staining for pMLC (A) and ppMLC (B), confocal max projections. The experiments were performed on six (pMLC) or three (ppMLC) donors, one is shown for each staining. Scale bar, 50 µm. (C,D) Quantification of pMLC (C) and ppMLC (D) fluorescence intensity from a representative donor.
Mean ± SD. Unpaired t-test, * $p < 0.05$, ** $p < 0.01$.

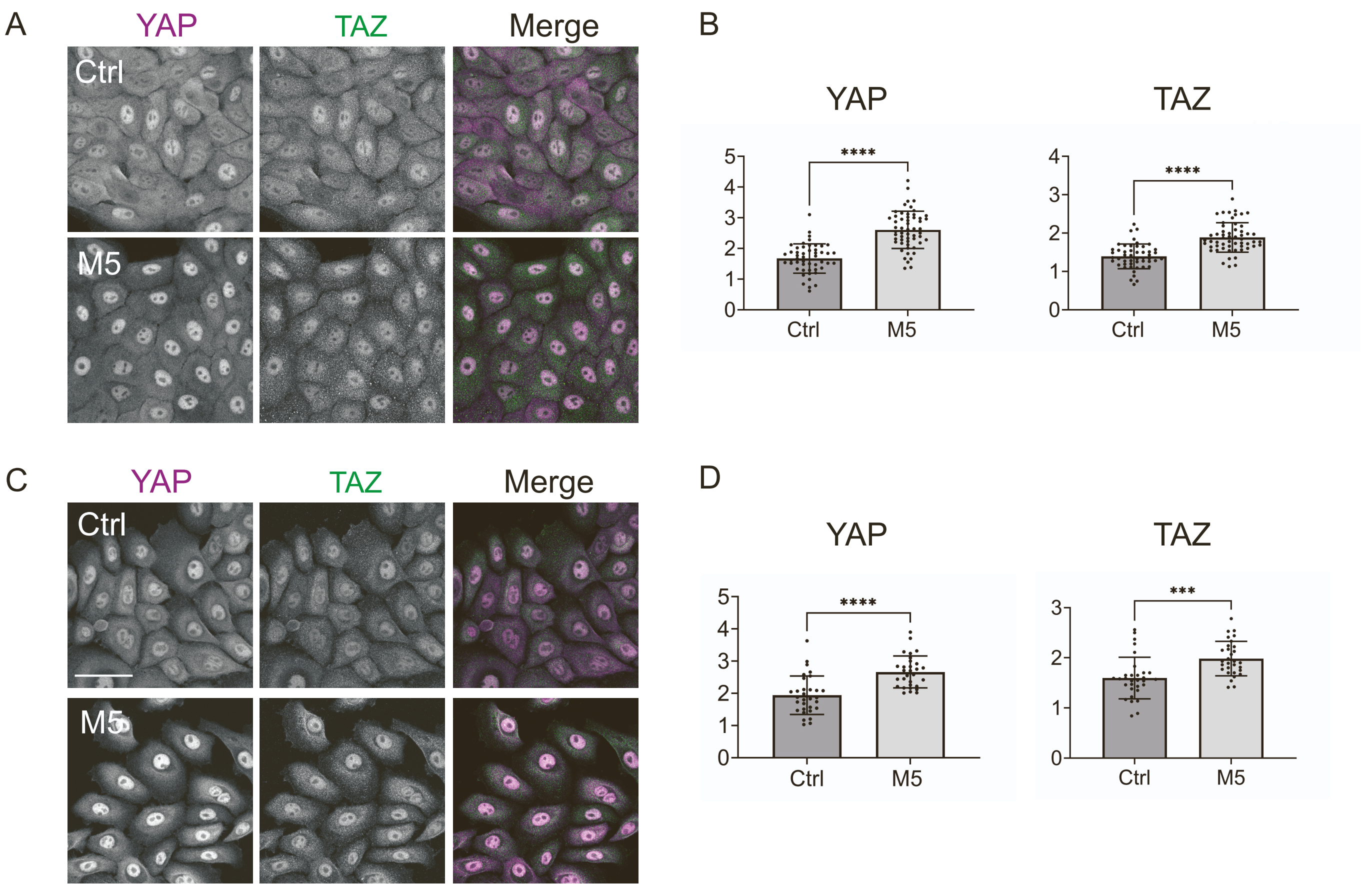

Figure S3. Related to Figure 3.

YAP and TAZ localization in N/TERT and in primary keratinocytes after 24 hours of M5 stimulation.

(A,C) Immunofluorescence staining of YAP and TAZ in N/TERT cells (A) and in primary keratinocytes (C), confocal max projections. Scale bar, 50 µm.

(B,D) Quantification for YAP and TAZ nucleus-to-cytoplasm ratios for N/TERT (B) and primary cells (D). For the N/TERT cells, another experiment similar to Figure 3A is shown. For the primary cells, one representative donor of three is shown.
Mean ± SD. Unpaired t-test, *** $p < 0.001$, **** $p < 0.0001$.

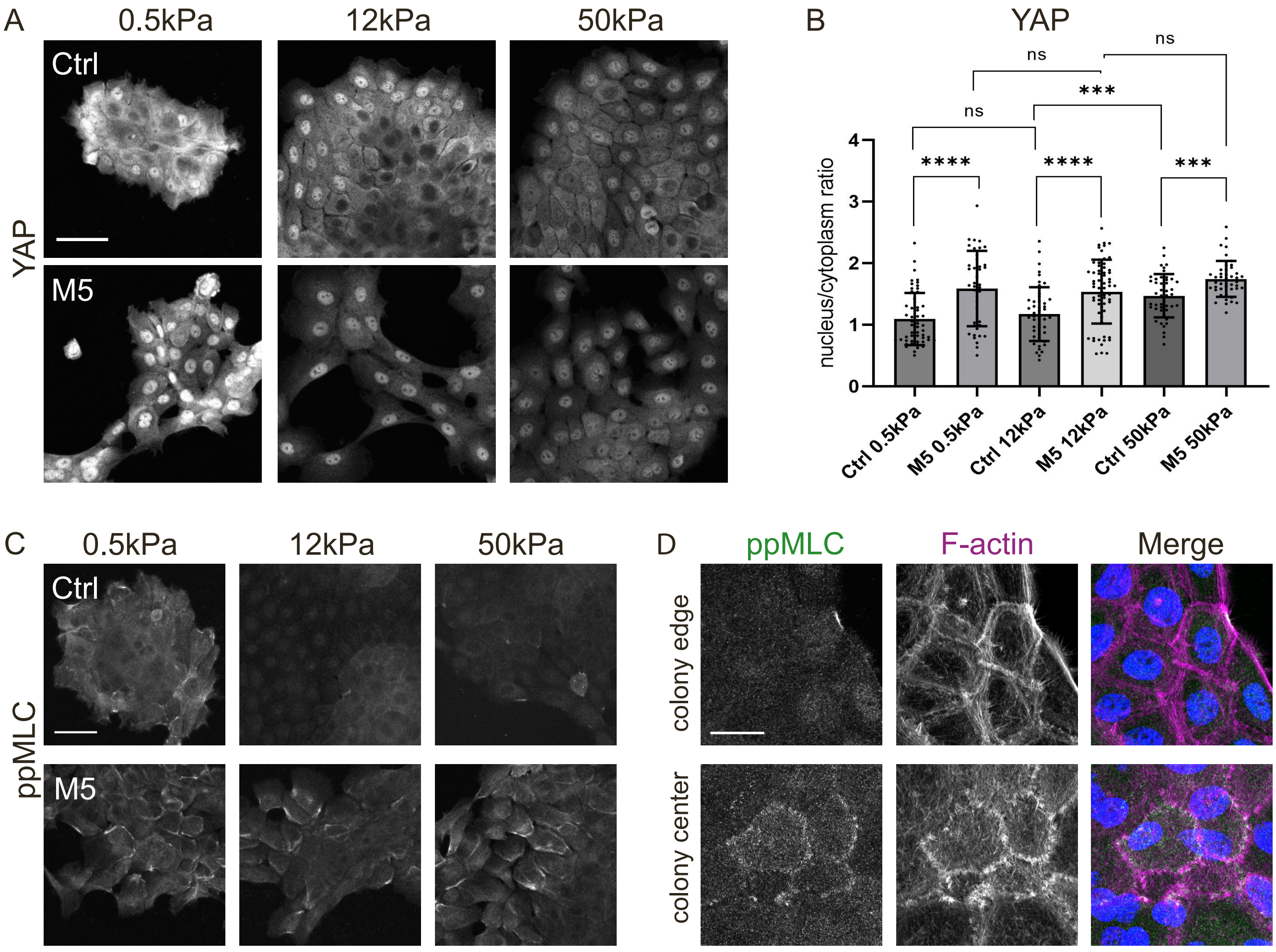

Figure S4. Related to Figure 3.

The effect of substrate stiffness on N/TERT keratinocytes in control and inflammatory conditions.

(A) Immunofluorescence staining for YAP at indicated substrate stiffness.

(B) Quantification of nucleus-to-cytoplasm ratio.
Mean ± SD. Unpaired t-test, *** $p < 0.001$, **** $p < 0.0001$.
One representative experiment of three is shown.

(C) Immunofluorescence staining for ppMLC at indicated substrate stiffness.

(D) Immunofluorescence staining for ppMLC in control cells on 12kPa.
Areas of a colony edge and center are shown at high magnification.

All images are confocal maximum projections. Scale bars, 50 μm (A,C), 20 μm (D).

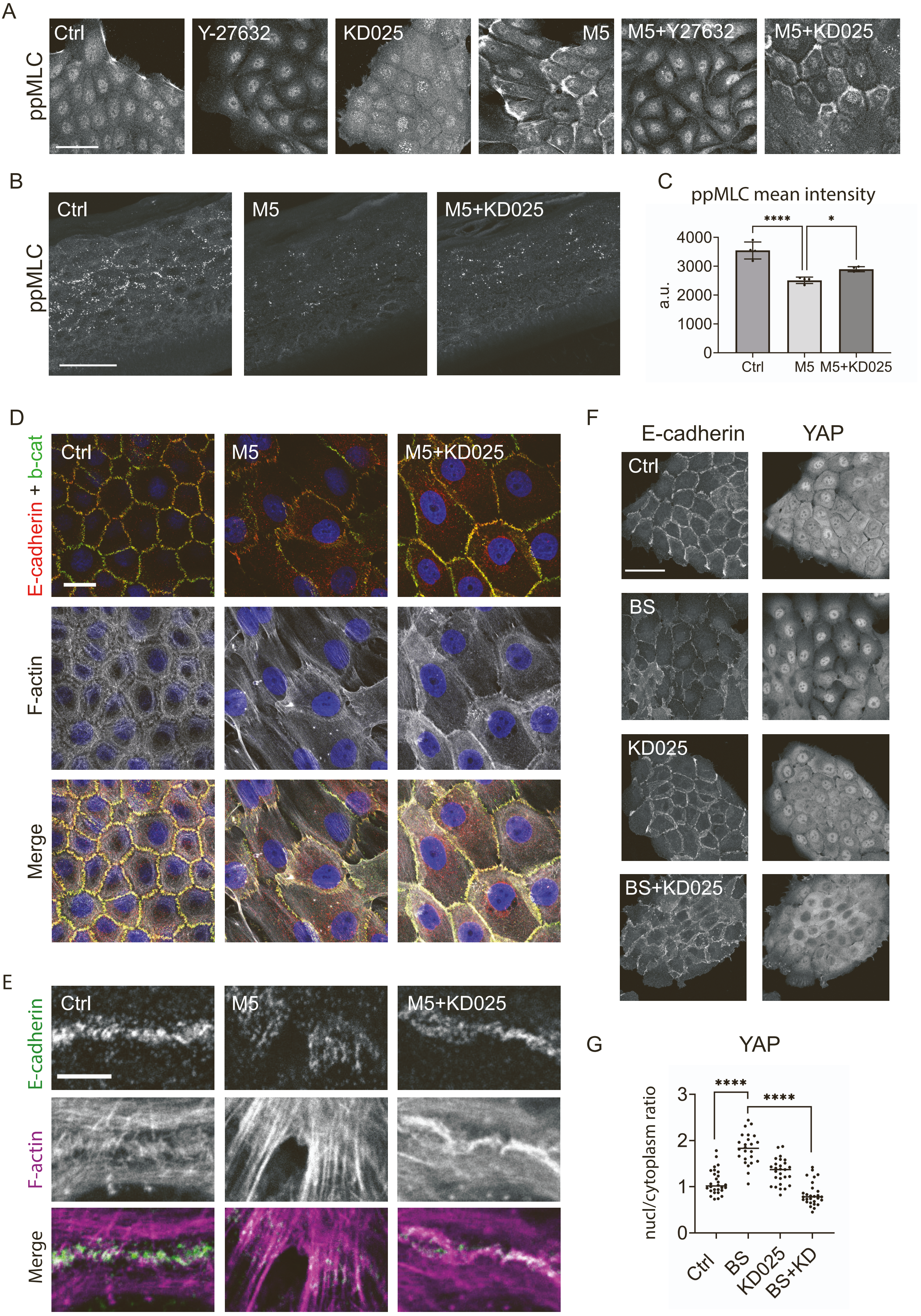
A
ppMLC
Ctrl
Y-27632
KD025
M5
M5+Y27632
M5+KD025
B
ppMLC
Ctrl
M5
M5+KD025
C
ppMLC mean intensity
****
*
4000
3000
2000
1000
0
a.u.
Ctrl
M5
M5+KD025
D
E-cadherin + b-cat
Ctrl
M5
M5+KD025
F-actin
Merge
E
E-cadherin
Ctrl
M5
M5+KD025
F-actin
Merge
F
E-cadherin
YAP
Ctrl
BS
KD025
BS+KD025
G
YAP
****
****
3
2
1
0
nucl/cytoplasm ratio
Ctrl
BS
KD025
BS+KD

Figure S5. Related to Figure 4.

The effect of ROCK1 and ROCK2 inhibition on the cytoskeletal organization in the treated N/TERT keratinocytes.

(A) Immunofluorescence staining of ppMLC in non-differentiated cells under indicated conditions. Confocal max projections.

(B) Immunofluorescence staining of ppMLC in RHEs from N/TERT under indicated conditions. Confocal max projections.

(C) Quantification of ppMLC fluorescence intensity from (B). Mean ± SD. Unpaired t-test, *** $p < 0.001$.

(D) Fluorescent staining of E-cadherin, β-catenin and F-actin in non-differentiated cells after 48 hours of stimulation, max projections from four apical confocal slices.

(E). Typical AJ phenotypes in indicated conditions: E-cadherin and actin cytoskeleton at the sites of AJs.

(F) Immunofluorescence staining of E-cadherin and YAP in indicated conditions after one hour treatment.

(G) Quantification of nucleus-to-cytoplasm ratio of YAP from the conditions shown in (F). Mean ± SD. Unpaired t-test, *** $p < 0.001$, **** $p < 0.0001$.

Scale bars, 50 µm (A,B,D,F), 5 µm (E).

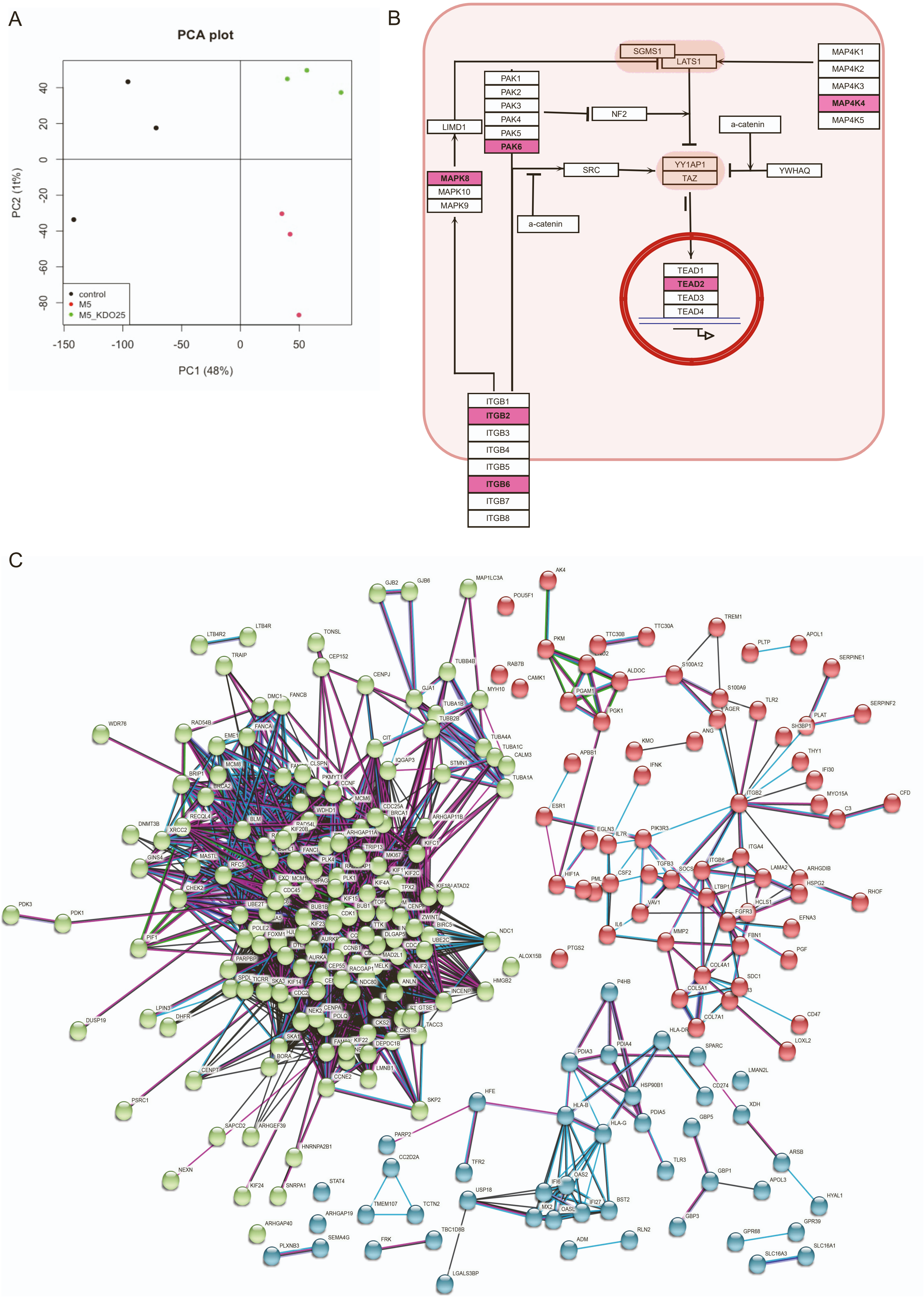
A
PCA plot
PC2 (11%)
PC1 (48%)
40
20
0
-20
-40
-60
-80
-150
-100
-50
0
50
control
M5
M5_KDO25
B
SGMS1
LATS1
MAP4K1
MAP4K2
MAP4K3
MAP4K4
MAP4K5
PAK1
PAK2
PAK3
PAK4
PAK5
PAK6
NF2
LIMD1
a-catenin
YY1AP1
TAZ
YWHAQ
SRC
MAPK8
MAPK10
MAPK9
a-catenin
TEAD1
TEAD2
TEAD3
TEAD4
ITGB1
ITGB2
ITGB3
ITGB4
ITGB5
ITGB6
ITGB7
ITGB8
C

Figure S6. Related to Figure 6.

Transcriptomic analysis of ROCK2 inhibition on M5-stimulated keratinocytes.

(A) PCA scatter plot of gene expression in N/TERT keratinocytes from three independent experiments stimulated with both M5 and KD025 (green dots), M5 alone (red dots) and control (black dots).

(B) Modified representation of mechanoregulation and pathology of YAP/TAZ via Hippo and non-Hippo mechanisms as reported in https://www.wikipathways.org/index.php/Pathway:WP4534, the genes downregulated by ROCK2 inhibition in M5-stimulated keratinocytes are shown in pink.

(C) String interaction analysis of the hub genes between “regulation of inflammatory mediators” and “cell cycle regulation” from the enrichment map of the DEGs between keratinocytes stimulated with both M5 and KD025 and M5 alone. Each node represents a DEG, mcl-clustered with an inflation parameter of three. Each cluster has a different color. Edges indicate various types of node interaction: experimentally determinate (pink line), from curated database (cyan line), gene neighbourhood (green line), gene co-occurrence (blue line), coexpression (black line) protein homology (violet line). All the genes were downregulated.
